# Application of Antioxidants from Agro-Industrial By-Products in Dairy Fat Systems: Oxidation Kinetics of Ghee and Active Packaging for Butter

**DOI:** 10.3390/foods15203736

**Published:** 2026-10-20

**Authors:** Itzel Yoali Hernández-Montesinos, David Fernando Carreón-Delgado, Liliana del Rocío Castro-López, Carolina Ramírez-López, Oxana Lazo Zamalloa

**Affiliations:** 1 Instituto Politécnico Nacional, Centro de Investigación en Biotecnología Aplicada, Carretera Estatal Santa Inés Tecuexcomac-Tepetitla, km 1.5, Tepetitla de Lardizábal 90700, Tlaxcala, Mexico; ihernandezm1308@alumno.ipn.mx (I.Y.H.-M.); dcarreond1300@alumno.ipn.mx (D.F.C.-D.); 2 Facultad de Enología y Gastronomía, Universidad Autónoma de Baja California, Carretera Transpeninsular Ensenada-Tijuana No. 3917, Colonia Playitas, Ensenada 22860, Baja California, Mexico; castro.liliana1@uabc.edu.mx

**Keywords:** agro-industrial by-products, pomace extracts, phenolic antioxidants, ghee, oxidative stability, active packaging

## Abstract

Apple, pear and grape pomaces are underexploited sources of phenolic compounds, potential natural alternatives to synthetic antioxidants. This study evaluated their cold-macerated (4 °C) extracts in ghee and in butter packaged in active chitosan films. Extracts were obtained under a 2^2^ factorial design (water or absolute ethanol; 24 or 48 h). Ethanol yielded higher total phenolic content (TPC) and antioxidant capacity than water, particularly in grape pomace. The 48 h ethanolic extracts of apple (AP-E), pear (PP-E) and grape (GP-E) pomaces were selected for their higher TPC and antioxidant capacity; GP-E showed an IC_50_ at least 94% lower than AP-E and PP-E in 2,2-diphenyl-1-picrylhydrazyl (DPPH) and 2,2′-azino-bis(3-ethylbenzothiazoline-6-sulfonic acid) (ABTS) assays. In ghee under accelerated storage (30, 45 and 60 °C, 35 days), extracts were compared to a synthetic Butylated Hydroxyanisole/Butylated hydroxytoluene (BHA/BHT) blend using oxidation kinetics and Arrhenius modeling. GP-E exhibited the lowest oxidation rate constants at all temperatures, significantly below BHA/BHT. PP-E did not differ significantly from the synthetic blend at 45 and 60 °C, and AP-E presented no significant effect at 60 °C. In butter wrapped in functionalized chitosan films (15 days storage at 21 and 30 °C), GP-E films produced the lowest oxidation values. PP-E films did not differ significantly from polyethylene control. These results suggest GP-E, and to a lesser extent PP-E, as a potential natural antioxidant for high-fat dairy matrices.

## 1. Introduction

The massive generation of organic solid waste constitutes one of the most critical environmental and socioeconomic challenges for the global agri-food system [1]. A substantial proportion of this waste corresponds to solid by-products generated during food processing, which have traditionally been disposed by landfilling, a practice associated with organic load leaching and uncontrolled greenhouse gas emissions [2]. Within the circular bioeconomy framework, cascading valorization through the recovery of high-value bioactive compounds has been proposed as an economical alternative to the current direct conversion of food agro-residues to biomass, biofuels or animal feed [3].

Among the recoverable fractions, phenolic compounds emerge as promising candidates for the development of new antioxidant additives for the food industry [4]. Their application for inhibiting lipid oxidation has acquired additional relevance given the increasing regulatory scrutiny of the synthetic antioxidants currently employed for this purpose, such as butylated hydroxyanisole (BHA) and butylated hydroxytoluene (BHT). In this vein, several jurisdictions have been established towards these compounds such as conservative daily intakes or restricted use [5], in addition to a growing consumer demand for clean-label formulations [6].

Grape (*Vitis vinifera* L.), apple (*Malus domestica* Borkh.), and pear (*Pyrus communis* L.) by-products represent promising matrices for natural antioxidant additives, due to the large volumes generated by the winemaking and juice industries, which produce pomace in the order of a quarter to a third of the processed fruit mass [7,8,9,10]. All three by-products have been reported to contain a diverse range of phenolic compounds. Grape pomace has been described as rich in anthocyanins and condensed tannins, which are responsible for its dark coloration and strong astringency, and to contain stilbenes such as resveratrol [11]. Apple pomace has been characterized by phloridzin, associated with a mildly sweet flavor [7,11]. Pear pomace, markedly less studied than the other two [8,12,13], has been reported to be dominated by chlorogenic acid and rutin [10,14,15].

Following the determination of antioxidant capacity in the free state, further consideration is whether this capacity can be extended beyond direct addition into the diverse forms in which natural antioxidants could potentially be delivered within food applications. One plausible source for application is ghee, since it represents an anhydrous fatty matrix and negligible moisture suitable for studying the capacity of phenolic extracts in the inhibition of its thermo-oxidative degradation, promptly to occur due to its very high fat content [16]. Lacking water and non-fat solids, ghee is microbiologically stable, so its shelf life is governed almost entirely by oxidative rancidity, which accelerates under thermal stress and leads to the accumulation of hydroperoxides and secondary oxidation products of documented physiological distress [17,18]. In addition, ghee is a commercially and culturally significant product for which oxidative stability constitutes a recognized industrial concern [16]. The antioxidants conventionally used for avoiding its oxidation (principally BHA and BHT) have been reported to lose protective efficacy at the elevated temperatures that are reached during its preparation and use, owing to volatilization and thermal decomposition [19].

In this vein, active packaging represents another possible vehicle for phenolic extracts, conferring antioxidant protection to the food surface through direct contact or controlled migration from the packaging matrix, in contrast to their direct incorporation as a functional ingredient within a solid lipidic matrix [20]. Among biodegradable film-forming polymers, chitosan offers well-documented film-forming capacity, biodegradability and compatibility with phenolic compounds through hydrogen bonding with its amino and hydroxyl groups [21]. Thus, the aim of the present work consisted of an exploratory assessment of the antioxidant potential of grape, apple, and pear pomace extracts across a range of application contexts, evaluating the oxidative stability and kinetic modeling of lipid degradation in ghee under accelerated storage conditions, and the protective performance of chitosan-based active films in delaying lipid oxidation in butter.

## 2. Materials and Methods

### 2.1. Reagents and Solvents

All chemical reagents and solvents used in this study were obtained from Sigma-Aldrich, Inc. (Toluca, Mexico) and J.T. Baker (Mexico City, Mexico), respectively.

### 2.2. Sampling

Apple (*Malus domestica*) and pear (*Pyrus communis*) pomaces were obtained as by-products of juice extraction of locally sourced fruits (Puebla, Mexico; cultivars not identified), processed unpeeled with a stainless-steel centrifugal juicer. Grape pomace (*Vitis vinifera* cv. Syrah, 2025 vintage) was obtained as a winemaking by-product from a local producer in Baja California, Mexico, and was collected once alcoholic fermentation of the must was completed and pressing had finished. All pomaces comprised peels, seeds and stems, and were air-dried at 40 °C for 18 h immediately after being obtained, ground, sieved through a 2 mm mesh, and stored in dark glass containers at 4 °C for no longer than one week before extraction.

### 2.3. Extract Preparation

Pomace extracts were obtained by cold maceration at 4 °C following a full factorial design (2^2^ = 4 treatment combinations, Table 1), with solvent type (distilled water or absolute ethanol, reagent grade) and extraction time (24 or 48 h) as fixed factors. Briefly, 10 g of dried pomace was mixed with 25 mL of the corresponding solvent in tightly closed amber glass flasks and kept under atmospheric conditions, without inert gas flushing. After the extraction period, extracts were filtered through Whatman No. 4 filter paper and stored in dark glass containers at 4 °C until further analysis.

### 2.4. Total Phenolic Content (TPC) of Pomace Extracts

For TPC, 100 µL of sample was mixed with 100 µL of Folin–Ciocalteu reagent (0.1 N) and 1.5 mL of distilled water and allowed to stand for 10 min. Subsequently, 300 µL of 20% *w*/*v* sodium carbonate (Na_2_CO_3_) solution were added and the mixture was incubated for 60 min in the dark at room temperature (22 ± 3 °C) [22]. After incubation, 200 µL aliquots of the reaction mixture was transferred to a 96-well microplate, and absorbance was measured at 765 nm using a Multiskan GO microplate spectrophotometer (Thermo Fisher Scientific, Waltham, MA, USA). Results were expressed as milligrams of gallic acid equivalents per gram of dry pomace (mg GAE/g), using the gallic acid calibration curve shown in Figure S1 of the Supplementary Material (linearity range 0–0.35 mg/mL; y = 2.795x + 0.015, R^2^ = 0.9969).

### 2.5. Antioxidant Capacity (AC) of Pomace Extracts

The antioxidant capacities were assessed using the 1,1-diphenyl-2-picrylhydrazyl (DPPH), 2,2-azino-bis(3-ethylbenzothiazoline-6-sulfonate) (ABTS) and the Ferric Reducing Antioxidant Power (FRAP) assays. For the DPPH assay, various concentrations of the sample (50 µL) were combined with DPPH ethanolic solution (150 µL, 90 µM) and incubated at 37 °C in the absence of light for 30 min. The absorbance was read at 517 nm [22]. For the ABTS assay, a stock solution of ABTS radical was prepared by mixing ABTS (7 mM) and potassium persulfate (2.45 mM) in equal volumes and allowing the mixture to incubate in darkness for 12 h. This stock solution was diluted with deionized water to achieve an absorbance of 0.70 ± 0.02 at 734 nm. Subsequently, different concentrations of the sample (10 µL) were mixed with diluted ABTS (190 µL) and incubated in darkness for 7 min. The absorbance was measured at 734 nm [23]. The radical scavenging percentage was determined by assessing the change in absorbance resulting from the reduction in DPPH and ABTS compared to the control.

For Trolox-Equivalent Antioxidant Capacity (TEAC) determination, all assays were also conducted using 6-hydroxy-2,5,7,8-tetramethylchroman-2-carboxylic acid (Trolox) as a standard to express results as micromoles of Trolox equivalents per gram of dry pomace (µmol TE/g), using Trolox calibration curves over a linearity range of 0–400 µmol/L (Figure S1, Supplementary Material). IC_50_ values, representing the concentration of extracts necessary to inhibit 50% of radicals, were determined by plotting extract concentrations (0–16% *v*/*v*) against radical scavenging percentages and adjusting the data to a three-parameter logistic dose–response regression model.

FRAP assay was employed to assess the antioxidant capacity of samples by measuring their ability to reduce ferric iron (Fe^3+^) to ferrous iron (Fe^2+^). Fresh FRAP reagent was prepared by mixing 300 mM acetate buffer (pH 3.6), 10 mM TPTZ (2,4,6-tripyridyl-s-triazine) in 40 mM HCl, and 20 mM FeCl_3_·6H_2_O in a 10:1:1 ratio. Ten microliters of sample were added to each well, followed by 300 µL of FRAP reagent. The mixture was incubated at 37 °C for 30 min and absorbance was measured at 593 nm [24]. Results were expressed as micromoles of ferrous sulfate equivalents per gram of dry pomace (µmol FeSO_4_/g), using a FeSO_4_ calibration curve over a linearity range of 0–1 mmol/L, as shown in Figure S1 of the Supplementary Material (y = 1.586x + 0.02, R^2^ = 0.9976).

### 2.6. Preparation of Ghee and Antioxidant Treatments

Commercial unsalted cow butter (without added antioxidants) was converted into ghee through a two-step heating process; first at 90 °C until complete separation of the aqueous phase from the milk solids, then by heating at 110 °C for 15 min to promote their browning, and finally these were removed by filtration through sterile gauze. The resulting anhydrous fat fraction was cooled to 50 °C before antioxidant addition to prevent thermal degradation of phenolic compounds.

In order to compare the residues under identical extraction and application conditions, working concentration of selected extracts based on their TPC and antioxidant capacity was set at twice the IC_50_ (expressed as % *v*/*v* of extract) of the least active extract. Prior to incorporation, the extracts were concentrated tenfold by solvent evaporation, and the concentrated extracts were incorporated individually into the ghee at a dose equivalent to 10% *v*/*v* of the original extract (i.e., 1 mL of concentrate, obtained from 10 mL of extract, per 100 mL of ghee), under continuous stirring (500 rpm, 5 min) to ensure homogeneous distribution.

Due to the fact that the extracts were applied on an equal-volume basis, the phenolic dose differed among treatments. From the TPC of each extract and the measured density of the ghee (0.896 g/mL at 50 °C), the nominal amount of phenolic compounds incorporated was 47.7, 61.3, and 875.8 mg GAE/kg of ghee for AP-E, PP-E, and GP-E, respectively. The dry solid content of the extracts was determined gravimetrically and used to express the extraction yield (dry extract solids per dry pomace, % *w*/*w*) and the dose of extract solids incorporated into the ghee (Table 2).

The control consisted of ghee without antioxidants, to which the same volume of absolute ethanol was added (vehicle control). The synthetic antioxidant blend (BHA/BHT, 1:1 *w*/*w*), included as a positive reference, was dissolved in the same volume of absolute ethanol (1 mL per 100 mL of ghee) and added at 200 mg/kg (100 mg/kg of each compound), the maximum total antioxidant content permitted in food by the US FDA (0.02% of the fat or oil content; 21 CFR 182.3169 and 182.3173) and the combined maximum set for ghee by the Codex General Standard for Food Additives (CXS 192-1995; food category 02.1.1), which also limits BHA to 175 mg/kg and BHT to 75 mg/kg.

To promote evaporation of the residual ethanol, all samples, including the vehicle control and the BHA/BHT treatment, were held at 50 °C for 1 h. Treatments were compared at the applied doses, and synthetic antioxidants at their maximum permitted level served as a regulatory benchmark rather than as a dose-matched reference.

### 2.7. Accelerated Oxidative Stability Testing and Sampling Design

Oxidative stability was evaluated using an accelerated shelf-life testing (ASLT) approach based on the Arrhenius model. Each treatment was distributed in cylindrical clear glass jars (125 mL capacity; 4.7 cm diameter × 12.1 cm height) filled with 20 g of ghee (approximately 22 mL), leaving a headspace of approximately 100 mL, and closed with metal screw caps. Jars were stored in the dark at 30, 45, and 60 °C in temperature-controlled ovens (±1 °C). Three independent jars (*n* = 3) per treatment and temperature were withdrawn at 0, 7, 14, 21, 28, and 35 days of storage and kept frozen (−20 °C) until analysis. Withdrawn jars were not returned to storage, so that each sample underwent a single freeze–thaw cycle.

### 2.8. Oxidative Stability Indicators

Rancidity and oil quality indicators were evaluated according to standardized methods of the American Oil Chemists’ Society (AOCS, 2017 [25]).

#### 2.8.1. Acid Value

The acid value (AV, mg KOH/g) was determined following AOCS method Ca 5a-40. Ten grams of sample were dissolved in a 1:1 *v*/*v* ethanol–diethyl ether mixture and titrated with 0.1 N potassium hydroxide (KOH) using phenolphthalein as an indicator. AV was calculated as:

(1) AV = (V × N × 56.1)/W

where V is the volume of KOH solution consumed (mL), N is the normality of the KOH solution (0.1 N), 56.1 is the molecular weight of KOH (g/mol), and W is the sample weight (g).

#### 2.8.2. Peroxide Value

The peroxide value (PV, meq O_2_/kg) was determined following AOCS method Cd 8-53. Briefly, 5 g of sample was dissolved in a glacial acetic acid–chloroform solution (3:2 *v*/*v*) and 0.5 mL of saturated potassium iodide solution was added. After 1 min reaction in the dark, the liberated iodine was titrated with 0.01 N sodium thiosulfate using starch solution as an indicator. PV was calculated as:

(2) PV = (V × N × 1000)/W

where V is the volume of sodium thiosulfate consumed (mL), N is the normality of the thiosulfate solution (0.01 N), and W is the sample weight (g).

#### 2.8.3. p-Anisidine Value

The p-anisidine value (p-AV) was determined following AOCS Official Method Cd 18-90. One gram of sample was dissolved in 25 mL of isooctane; 5 mL of this solution was mixed with 1 mL of p-anisidine solution in glacial acetic acid (0.25% *w*/*v*) and incubated at 25 °C for 10 min in the dark. Absorbance was measured at 350 nm against a blank containing isooctane and p-anisidine reagent without a sample. The p-AV was calculated as:

(3) p-AV = 25 × (1.2 × A_2_ − A_1_)/W

where A_1_ is the absorbance of the unreacted sample solution, A_2_ is the absorbance of the reacted mixture, 1.2 corrects for the volume increase upon reagent addition, 25 is the standard method factor, and W is the sample weight (g).

#### 2.8.4. Total Oxidation Index

The total oxidation index (TOTOX) was calculated as:

(4) TOTOX = 2 × PV + p-AV

TOTOX integrates both primary (hydroperoxides) and secondary (carbonyl compounds) oxidation products, providing a comprehensive index of cumulative lipid oxidation.

#### 2.8.5. Color Analysis

The color values of ghee samples were evaluated with a Precise Colorimeter (CS-210, CHN Spec Technology Co., Ltd., Hangzhou, China). Three measurements were taken for each sample, and the average of three readings was calculated for each measurement [26]. Collected data included lightness values (L*), redness values (a*), and yellowness values (b*). The overall color difference (Δ*E*) was calculated using the following Equation (5).

(5) $$∆E=\sqrt{{\left(L_{0}^{*}-L^{*}\right)}^{2}+{\left(a_{0}^{*}-a^{*}\right)}^{2}+{\left(b_{0}^{*}-b^{*}\right)}^{2}}$$

where L*_0_, a*_0_, and b*_0_ are the initial values and L*, a*, and b* are the values of the sample at the evaluated time points. ΔE was calculated for each replicate relative to its own day-0 reading.

### 2.9. Oxidation Kinetics and Arrhenius Modeling

For each treatment and temperature (30, 45, and 60 °C), the time-course data of TOTOX were fitted to zero-order and first-order kinetic models to identify the best descriptor of oxidation progression:

(6) Zero-order: C(t) = C_0_ + *k*t

(7) First-order: ln C(t) = ln C_0_ + *k*t

where C(t) is the TOTOX value at time t (days), C_0_ is the initial TOTOX value, and *k* is the apparent rate constant (day^−1^ for first order; TOTOX·day^−1^ for zero-order). Model selection was based on the coefficient of determination (R^2^) and the root mean square error (RMSE). The model yielding the highest R^2^ and lowest RMSE was selected for each treatment.

The temperature dependence of the rate constants was described by the Arrhenius equation:

(8) ln(*k*) = ln(A) − E_a_/(R·T)

where *k* is the rate constant at absolute temperature T (K), A is the pre-exponential frequency factor, E_a_ is the activation energy (kJ/mol), and R is the universal gas constant (8.314 J/mol·K). E_a_ and ln(A) were estimated by linear regression of ln(*k*) against 1/T for each treatment. To carry the variability of the rate constants into E_a_, the model was fitted independently to each replicate series (three temperature-specific k values per replicate); its 95% confidence interval was obtained from a pooled regression of the nine replicate-level k values of each treatment (7 degrees of freedom). As only three temperatures were evaluated, the E_a_ values are considered apparent estimates. A higher E_a_ indicates greater temperature sensitivity of oxidation, whereas a lower k indicates a slower oxidation rate under the tested conditions.

### 2.10. Application for Active Packaging

#### 2.10.1. Preparation and Functionalization of Chitosan-Based Edible Films

Chitosan-based films were prepared by casting. Medium molecular weight chitosan (Sigma-Aldrich, St. Louis, MO, USA; degree of deacetylation 75%) was dissolved at 2% (*w*/*v*) in 1% (*v*/*v*) acetic acid at 75 °C under continuous stirring for 1 h; the temperature was then lowered to 40 °C and stirring continued for a further hour to ensure complete dissolution. Glycerol (1% *w*/*v*) was incorporated as plasticizer, followed by the selected extracts (AP-E, PP-E, and GP-E), concentrated as described in Section 2.6 and added at a dose equivalent to 10% *v*/*v* of the original extract (1 mL of concentrate per 100 mL of film-forming solution). The homogenized film-forming solutions were cast onto flat-bottomed silicone molds (15 cm × 25 cm) at a constant casting ratio of 90 mL per mold to control final film thickness. Molds were placed on a leveled surface in a forced-convection oven and dried at 32 °C for at least 48 h until constant weight (resulting in average thickness of 107.53 ± 6.54 µm). The dried films were peeled from the molds and stored at room temperature in hermetically sealed plastic bags until use. Control films were prepared identically, with the same volume of absolute ethanol added without extract.

#### 2.10.2. Application of Chitosan-Based Films for Active Packaging in a Butter Model

Blocks of commercial unsalted cow butter without added antioxidants (20 g; approximately 3.25 cm × 3.25 cm × 2 cm) were wrapped directly between two pieces of film (6.25 cm × 7.5 cm), and the four edges were heat-sealed with an impulse sealer. Unfunctionalized chitosan film (control film) and commercial polyethylene Ziplock bags were used as references. Samples were stored at room temperature (RT, 21 °C) or in an oven at 30 °C (oven) for 15 days, and AV, PV, p-AV and TOTOX were determined at days 0 and 15 (Section 2.8).

### 2.11. Fourier Transform Infrared Spectroscopy (FT-IR)

Fourier Transform Infrared Spectroscopy (FT-IR) was employed to characterize the extracts, chitosan, and films. Measurements were performed using a Bruker^®^ Vertex 70 FT-IR spectrometer (Ettlingen, Germany) in ATR mode, covering a range of 4000–400 cm^−1^, at a nominal resolution of 4 cm^−1^. Each sample was analyzed in triplicate to ensure reproducibility of the spectral data.

### 2.12. Statistical Analysis

All experiments were performed in triplicate (n = 3), except for the data in Table 3 and Figure 1 (n = 4). Results are expressed as mean ± SD, and individual replicate values are available in Tables S1–S6 of the Supplementary Material. Differences among treatments were evaluated by ANOVA followed by Tukey’s HSD post hoc test (α = 0.05), and differences relative to the initial value were assessed using Dunnett’s post hoc test (α = 0.05). Normality and homogeneity of variance were verified using the Shapiro–Wilk and Levene’s tests, respectively; where Levene’s test indicated heteroscedasticity, Welch’s ANOVA was used to confirm the treatment effect followed by Games–Howell post hoc test (α = 0.05). Statistical analyses were performed using Minitab 19 for Windows (Minitab Inc., State College, PA, USA). Graphs and IC_50_ calculations were performed using GraphPad Prism 9 for Windows (GraphPad Software, San Diego, CA, USA).

## 3. Results

### 3.1. Total Phenolic Content (TPC) and Antioxidant Capacity (DPPH, ABTS, and FRAP) of Pomace Extracts

#### Factorial Design

A two-way ANOVA confirmed solvent and extraction time as significant factors (*p* < 0.05) for all pomace source–parameter combinations, with the solvent effect consistently showing the largest F-values. Model fit was high (R^2^ ≥ 0.84), and a significant solvent × time interaction was detected mainly for grape pomace across all four parameters and, to a lesser extent, for pear (TPC) and apple (TEAC-ABTS and FRAP). For all three pomace sources, ethanol yielded higher TPC and antioxidant capacity than water at both extraction times (Table 3), with the magnitude of the solvent effect being higher for grape than for apple or pear pomace. Extending the extraction time from 24 to 48 h increased both parameters, reaching statistical significance mainly in the ethanolic extracts.

Pooling across solvent and time to compare sources directly (Table 4), Welch’s ANOVA with Games–Howell post hoc showed that grape pomace extracts had significantly higher TPC and antioxidant capacity than apple and pear extracts (*p* ≤ 0.001), which did not differ significantly from each other in TPC, TEAC-ABTS or FRAP. Absolute ethanol and a 48 h extraction time were therefore selected for obtaining the extracts used in subsequent characterization and application.

### 3.2. Characterization of the Selected Extracts for Application Development

#### 3.2.1. Functional Characterization

TPC and antioxidant capacity of the three selected 48 h ethanolic extracts (AP-E, PP-E, and GP-E) are shown in Figure 1A. The extracts showed a visible color gradient consistent with their phenolic content, from pale yellow in AP-E to light amber in PP-E and dark reddish-brown in GP-E. Based on the dry solids content of each extract (8.19 ± 0.71, 8.40 ± 0.69 and 16.63 ± 0.98 mg/mL), the gravimetric extraction yields were 2.05 ± 0.18, 2.10 ± 0.17 and 4.16 ± 0.25% *w*/*w* of dry pomace for AP-E, PP-E and GP-E, respectively. Dose–response curves fitted to a three-parameter model (R^2^ ≥ 0.98) yielded IC_50_ values for DPPH and ABTS radical scavenging (Figure 1B,C). GP-E showed an IC_50_ at least 94% lower than AP-E and PP-E for both assays, indicating higher radical scavenging capacity per unit volume of extract. AP-E and PP-E displayed comparable IC_50_ values for both radicals, in line with their statistically similar TPC and antioxidant capacity, confirming GP-E as the extract with the highest antioxidant capacity among the three. Extract concentrations above their IC_50_ (10% *v*/*v*) were therefore selected for subsequent application in butter models.

#### 3.2.2. FT-IR Characterization of Selected Extracts

The FT-IR spectra of the three extracts (Figure 2) shared a common set of absorption bands consistent with their botanical origin, together with source-dependent differences in band position and relative resolution. The O–H stretching band appeared at 3358 cm^−1^ in AP-E and PP-E and at 3335 cm^−1^ in GP-E, arising from the superposition of hydroxyl groups of phenolic compounds, carbohydrates and residual water [27,28]. The asymmetric and symmetric C–H stretching doublet of aliphatic –CH_3_ and –CH_2_ groups occurred at 2978 and 2899 cm^−1^ in AP-E and PP-E, and at 2974 and 2884 cm^−1^ in GP-E [27,28,29]. In the fingerprint region, all three extracts displayed the C–O–C glycosidic band at 1086–1088 cm^−1^ and an intense C–O stretching band at 1043–1045 cm^−1^, the dominant feature of every spectrum, assigned to water-soluble polysaccharides and glycosylated phenolic structures [27,29], together with the out-of-plane C–H deformation of the pyranoside ring at 878–880 cm^−1^ [27]. Two further bands were identified in the three extracts as follows: 1649 cm^−1^, assignable to aromatic ring skeletal vibration and carboxylate stretching overlapping with conjugated flavonoid C=C stretching [27,28], and 1381 cm^−1^ in AP-E and PP-E (1379 cm^−1^ in GP-E), assignable to in-plane bending of phenolic O–H groups [29,30].

### 3.3. Oxidative Stability of Ghee Under Accelerated Storage Conditions

#### 3.3.1. Oxidation Kinetics and Arrhenius Modeling

AV, PV and p-AV were monitored over 35 days at 30, 45 and 60 °C. All three increased progressively across treatments and temperatures, with significant effects of treatment, temperature and their interaction with storage time (three-way ANOVA, *p* < 0.05).

Table 5 summarizes the results on day 35, when treatment differences were most pronounced: CONTROL reached the highest values for all three parameters at every temperature and GP-E the lowest. Relative to initial values, markers increased by approximately 3- to 7-fold in CONTROL across the 30–60 °C range and by 1.5- to 3-fold in GP-E. PP-E and BHT/BHA showed comparable intermediate increases (2- to 4-fold) and did not differ significantly from each other in any parameter or temperature. AP-E differed significantly from CONTROL at 30 °C for AV and PV but not at 45 or 60 °C, where its fold-change (6- to 7-fold) approached that of CONTROL.

To integrate the contribution of both primary and secondary oxidation products into an overall measure, the combined oxidation index (TOTOX) was calculated for each treatment and temperature (Figure 3A). Linearization of TOTOX as in Ln(TOTOX) vs. storage time yielded highly linear fits (R^2^ ≥ 0.93; Figure 3B), consistent with first-order kinetics.

Rate constants (*k*) increased with temperature for every treatment, with GP-E lowest and CONTROL highest at all three temperatures (Tukey’s HSD, *p* < 0.05), consistent with the ranking observed for the individual markers. Treatment separation narrowed as temperature increased, with PP-E and BHT/BHA no longer differing significantly at 45 and 60 °C, and CONTROL and AP-E showing no significant difference at 60 °C.

While *k* reflects how effectively each treatment protects against oxidation, the activation energy (Ea) reflects how temperature-sensitive that protection is. Arrhenius analysis (Figure 4) yielded activation energies that did not differ among antioxidant treatments (Tukey’s HSD). Comparison against CONTROL (15.44 kJ/mol) showed significantly higher Ea for BHT/BHA (21.84 kJ/mol), PP-E (19.97 kJ/mol), and GP-E (21.79 kJ/mol) while AP-E (18.55 kJ/mol) did not differ significantly (Dunnett’s test, *p* < 0.05).

#### 3.3.2. Surface Color Change During Accelerated Storage

Treatments significantly affected total color difference at every temperature and sampling day (Tukey’s HSD, *p* < 0.05). Color change increased with storage time at the three temperatures, with an identical treatment ranking throughout: GP-E < PP-E < BHA/BHT < AP-E (Figure 5). At day 35, GP-E showed the smallest change, corresponding to 55.3%, 46.1% and 54.8% inhibition relative to the control at 30, 45 and 60 °C; it was the only treatment differing significantly from BHA/BHT at all three temperatures, and its inhibitory capacity was unaffected by increasing temperature. PP-E was the second most effective, with inhibition decreasing from 46.9% at 30 °C to 30.6% at 45 °C and 26.2% at 60 °C, differing from GP-E only at 60 °C while remaining different from the control at all temperatures. BHA/BHT inhibited color change by 15.2–23.1% without reaching significance against the control at any temperature, and AP-E did not inhibit color change under any condition.

Decomposition of ΔE at day 35 showed that color change was almost entirely attributable to increasing yellowness, with Δb* accounting for 97.1% of ΔE^2^ against 2.0% for ΔL* and 0.8% for Δa* (Figure 6). Mean b* increased by 25.7, 30.3 and 37.9 units at 30, 45 and 60 °C, and initial b* values differed among treatments as a consequence of the intrinsic color of the extracts. Lightness decreased with storage temperature (−3.41 at 45 °C, −7.24 at 60 °C), concentrated at the final sampling point of the highest temperature; the combination of decreasing L*, positive a* and high b* is consistent with the onset of non-enzymatic browning in the least protected samples.

### 3.4. Functionalized Chitosan Films in Active Packaging (Butter Model)

#### 3.4.1. Performance of Functionalized Chitosan-Based Films

For all four oxidation indices, the control film showed the highest values at both storage temperatures and GP-E Film the lowest, with AP-E Film, PP-E Film and Ziplock bag at intermediate levels (Table 6, Figure 7B). PP-E Film and Ziplock bag did not differ significantly in any parameter or storage condition (*p* < 0.05), whereas AP-E Film was significantly higher than both. Storage temperature significantly affected every treatment and parameter, with all oven values higher than their RT counterparts.

A significant Treatment × Storage condition interaction was found for AV and p-AV, whereas PV showed a weaker interaction and TOTOX showed none, indicating that the relative ranking for total oxidation remained stable regardless of storage temperature. After 15 days, all treatments retained a pale-yellow color at RT, whereas under oven conditions both films and butter showed a noticeable color change, most evident in AP-E Film and PP-E Film (Figure 7A). Nearly all treatment–condition combinations showed significant increases in oxidative markers relative to the day-0 samples.

#### 3.4.2. FT-IR Characterization of Chitosan-Based Edible Films

Chitosan displayed its characteristic bands (Figure 8): O–H/N–H stretching at 3360 cm^−1^, C–H stretching at 2870 cm^−1^, Amide I at 1651 cm^−1^, Amide II at 1558 cm^−1^, glycosidic C–O–C at 1150 cm^−1^, pyranose C–O at 1026 cm^−1^, and a discrete low-intensity band at 895 cm^−1^ assigned to the β anomeric configuration. Incorporation of glycerol displaced the O–H/N–H band to 3285 cm^−1^ in Control-F (Δ = −75 cm^−1^), while the C–O–C and C–O bands remained close to their native positions. In the 780–1000 cm^−1^ region, Control-F displayed a broad absorption envelope of markedly higher intensity than the 895 cm^−1^ band of chitosan, with local maxima at 851 and 924 cm^−1^, within which no discrete feature was distinguishable at 895 cm^−1^.

The three functionalized films retained the C–O–C and C–O skeletal bands at essentially unchanged positions (1152 and 1024–1028 cm^−1^) and displayed the same 780–1000 cm^−1^ envelope described for Control-F. No band was detected in the 1720–1760 cm^−1^ or 1255–1275 cm^−1^ intervals in any system. Amide I, at 1651 cm^−1^ in chitosan, was displaced to 1601 cm^−1^ in Control-F and occurred at intermediate positions in the functionalized films, appearing as an unresolved shoulder in AP-F and GP-F and as a discrete band at 1649 cm^−1^ in PP-F. Amide II was displaced to 1555, 1557 and 1560 cm^−1^ in AP-F, PP-F and GP-F respectively.

## 4. Discussion

### 4.1. Extraction Efficiency and Characterization of the Selected Extracts for Application Development

Ethanol produced higher TPC and antioxidant capacity than water in the three pomaces at both extraction times. This is consistent with the reduction in the dielectric constant of the medium, which shifts solvent polarity towards the range favoring solubilization and diffusion of phenolic compounds [10], and with the reported interaction of ethanol with the phospholipid bilayer of plant cell membranes, increasing permeability and reducing resistance to mass transfer [9,10]. The solvent effect was consistently larger for the antioxidant capacity assays than for TPC, in agreement with reports that aqueous extraction may yield high gross masses through dissolution of sugars, pectins and minerals, while the specific phenolic concentration and radical scavenging capacity remain lower [4,31].

The magnitude of this effect differed among pomaces, which may reflect differences in the composition of the available phenolic fraction. Grape pomace has been described as rich in lipophilic and intermediate-polarity compounds such as stilbenes and condensed tannins [32,33], whose recovery is favored by higher ethanol proportions required to overcome steric solubility constraints [9,34], whereas apple and pear pomaces have been described as rich in glycosylated fractions and polar phenolic acids highly soluble in aqueous media [7,10,12]; this would be consistent with the recovery obtained with water being closer to that obtained with ethanol in these two matrices.

Extending extraction from 24 to 48 h increased both parameters numerically in all cases, reaching significance mainly in the ethanolic extracts. Solid–liquid extraction has been described as a two-stage process comprising a rapid convective phase followed by a slower diffusive phase that governs the approach to equilibrium [35]; the persistence of a positive time effect at 48 h is consistent with the low extraction temperature employed, which slows diffusion and delays saturation relative to thermally assisted protocols [9]. Prolonged contact is not, however, universally beneficial: losses have been reported beyond an optimum through spontaneous oxidation and polymerization of phenolic monomers into insoluble species, as documented in grape pomace macerated in ethanol at 25 °C between 10 and 30 days [36], and at shorter times under ultrasonically assisted conditions [10].

For the extracts selected for application (absolute ethanol, 48 h), the gravimetric yields were low (2.05–4.16% *w*/*w*), as expected for unstirred maceration at 4 °C, where mass transfer is limited [9,34]. The TPC obtained for GP-E with absolute ethanol at 48 h falls within the broad range reported for this residue, spanning from 4.07 mg GAE/g under an optimized hydroalcoholic mixture at ambient temperature [9] through 25 mg GAE/g for Syrah pomace with ethanol:water 1:1 at 50 °C [37] to 85.63 mg GAE/g under pressurized liquid extraction at 40–100 °C [35], a dispersion governed largely by extraction temperature and cultivar [9,27]. For AP-E, in contrast to the present results, an aqueous extract has been reported to exceed an ethanolic one in both phenolic recovery and radical scavenging capacity [38], a discrepancy that has been attributed to the high hydrophilic affinity of the glycosylated flavonols and free hydroxycinnamic acids reported as dominant in this matrix [7], reinforcing the hypothesis that the phenolic fraction of these residues is efficiently recovered in aqueous media and that the advantage of a less polar solvent is modest and may not be reproduced under all extraction conditions.

The IC_50_ of GP-E was at least 94% lower than those of AP-E and PP-E in both assays, a difference larger than that observed for TPC alone, therefore indicating higher radical scavenging potency per unit of extract relative to its phenolic content. For grape pomace specifically, IC50 values as low as 33.5 µg/mL have been reported when expressed relative to the mass of a lyophilized extract [39]. The three assays yielded the same ranking of the extracts and the same response to solvent and extraction time in all three pomaces, despite relying on different chemical principles. The FRAP assay proceeds exclusively through a single electron transfer (SET) mechanism, whereas the DPPH and ABTS assays evaluate radical scavenging activity and may operate through hydrogen atom transfer (HAT), SET, or mixed mechanisms, depending on the structure of the antioxidant and on the reaction medium [40]. This agreement across assays operating through different mechanisms indicates that the superior antioxidant capacity of GP-E is not contingent upon the analytical method, and that the phenolic fractions recovered are active both as electron donors and as radical scavengers.

The differences among pomaces cannot be attributed to the botanical source alone. The grape pomace had undergone maceration and alcoholic fermentation, during which part of its phenolic compounds is transferred to the wine, whereas the apple and pear pomaces were unfermented residues of juice extraction.

Spectroscopic characterization of fruit pomace phenolic extracts is largely restricted to grape pomace [27,29,39,41], with partial characterization for apple by-products and apple pomace flour [28,41,42] and, to the best of our knowledge, none for pear pomace extract. The band positions observed here are in close agreement with those reported for phenolic extracts of fruit by-products obtained by different procedures and solvents, including grape pomace extracts obtained with 70% ethanol at room temperature and native solid samples analyzed after oven drying at 50 °C [29], apple pomace extracts recovered using multi-solvent systems [28], grape and apple pomace extracts isolated with 50% acetone or pure ethanol [41], and sour cherry pomace extracts obtained through microwave- and ultrasound-assisted extraction [43]. These results suggest that 48 h cold ethanolic maceration yields spectral profiles which are comparable to those obtained by methods involving high temperatures and assisted techniques.

The O–H stretching band appeared at a lower wavenumber in GP-E (3335 cm^−1^) than in AP-E and PP-E (3358 cm^−1^). This region arises from the broad superposition of hydroxyl groups belonging to diverse phenolic compounds, co-extracted carbohydrates, and aliphatic alcohols [27,28]. Regarding the structural informativeness of the 3300–3700 cm^−1^ region, in some chemometric models for phenolic content the interval has demonstrated high predictive weight [42,44], while other studies exclude it due to its lack of specificity for individual phenolic classes [45].

The bands at 1379–1381 cm^−1^ have been attributed to in-plane bending of phenolic O–H groups [29,30]. The most prominent band in this region was observed in the GP-E spectrum, qualitatively consistent with its higher phenolic content. Meanwhile, the neighboring 1649 cm^−1^ band, to which carboxylate and aromatic C=C stretching both contribute [27,28], was comparatively less prominent in that same spectrum.

### 4.2. Oxidative Stability of Ghee Under Accelerated Storage Conditions

Oxidation kinetics were characterized across 30, 45 and 60 °C to describe the oxidative behavior of ghee under both realistic and accelerated storage. Kinetic modeling of this kind has been applied across a wide range of lipid matrices and temperature intervals, with first-order models fitted to peroxide value in butter oil from milk and yogurt across 60–80 °C [46] and to pomegranate seed oil across 30–50 °C [47], and zero-order models applied to vegetable oils stored at 35–55 °C [48], indicating that the appropriate reaction order is matrix- and condition-dependent rather than universal. In the present work, linearization of TOTOX yielded high coefficients of determination at all three temperatures (R^2^ ≥ 0.93), indicating that an apparent first-order model provides an adequate empirical description of the data, without establishing the underlying reaction mechanism. Ghee is described as a matrix of comparatively high intrinsic oxidative stability, attributable to the following three concurrent factors: a fatty acid profile in which saturated species account for 65–75% of the total [49,50], with a correspondingly low density of bis-allylic positions susceptible to radical attack [51]; a moisture content below 0.5% *w*/*w* that limits hydrolytic lipolysis and precludes microbial growth [17]; and native lipophilic antioxidants, chiefly tocopherols and carotenoids, acting as reducing agents and radical scavengers [17,46]. Its natural shelf life has nevertheless been described as shorter than that of several vegetable oils, owing to its comparatively low content of these native antioxidants [49]. Reported shelf-life endpoints for unprotected ghee extend across 180 days at 25 °C [30], 90 days at 50 °C [52] and 8 to 10 days at 80 °C [53], with pronounced acceleration of peroxide and TBA accumulation documented from day 9 onwards at 60 °C and deterioration by day 21 [54]; the values obtained in the present CONTROL after 35 days at 60 °C therefore fall within the range expected for an unprotected sample near the upper end of an accelerated regime.

Rate constants ranked consistently across all three temperatures, with GP-E lowest and CONTROL highest, reproducing the ordering obtained for the individual oxidation indices and for the antioxidant capacity of the three extracts. At the applied doses, PP-E (0.094% *w*/*w* of dry extract) did not differ significantly from the synthetic BHA/BHT blend (0.02% *w*/*w*) at 45 and 60 °C, and GP-E (0.186% *w*/*w*) showed the lowest rate constants. Since the treatments were not dose-matched, these results identify PP-E and GP-E as promising candidates as natural alternatives.

Comparable outcomes have been reported in ghee under similarly non-equivalent dosing, with rosemary extract at 0.1% *w*/*w* giving an induction period statistically identical to BHA at 0.02% [49], and microencapsulated galgal peel essential oil at 1.5% *w*/*w* matching BHA at 0.02% over 180 days at 25 °C [30]; in bulk oils, combined grape pomace extracts at 30% *v*/*v* performed similarly to BHT at 0.02% *w*/*w* [55].

GP-E lowered the rate constant by 52–61% relative to CONTROL across the three temperatures, BHA/BHT by 32–46% and PP-E by 29–39%, whereas AP-E lowered it by only 3–13%. Since under first-order kinetics the time required to reach a given TOTOX level is inversely proportional to k, these reductions correspond at 30 °C to TOTOX doubling times of 55 days for GP-E, 40 for BHA/BHT and 35 for PP-E, compared with 21 days for CONTROL. The rate constant of GP-E remained about 30% below that of BHA/BHT at all three temperatures, and the k of AP-E converged with that of CONTROL as temperature increased. The rate constants and doubling times reported here describe relative oxidation rates under the conditions tested; they were not validated against real-time storage and should not be taken as shelf-life predictions.

The lower rate constants observed for the treated samples indicate a consistent protective effect, while the higher Ea obtained for BHT/BHA, PP-E and GP-E indicates that this protection is comparatively more temperature-dependent than the intrinsic oxidation process of unprotected ghee, being more pronounced under mild storage conditions and attenuated as temperature rises. This pattern is consistent with several studies reporting an increase in the apparent activation energy of lipid oxidation upon antioxidant addition, documented in chia oil with ascorbyl palmitate and plant extracts, where the increase followed the order of effectiveness of each additive and ineffective antioxidants gave values below the control [56], as well as in pomegranate seed oil upon natural enrichment in phenolics and carotenoids [47]. The opposite direction has nevertheless been reported in a Rancimat-based study on perilla oil with rosemary extract, where a lower Ea was interpreted as indicating a system less sensitive to temperature fluctuation [57]. This behavior is consistent with the thermolability of both synthetic and phenolic antioxidants.

Synthetic antioxidants are reported to be unstable at elevated temperatures, being lost through volatilization and chemical transformation into inactive derivatives [5,58]. Phenolic compounds degrade analogously through cleavage and polymerization of their hydroxyl groups, with direct heating of ghee measurably reducing the radical scavenging capacity of added plant extracts [17,18]. Since antioxidant depletion has a higher intrinsic activation energy than the autoxidation it retards, the two processes are superimposed in the apparent Ea of a protected system, so that the reserve of active compounds collapses faster than the underlying oxidation accelerates as temperature rises [59,60]. This offers a plausible mechanistic basis for the elevated Ea observed in the protected treatments without implying any loss of their protective advantage over CONTROL and is consistent with the narrowing of treatment differences at the highest temperature, a pattern documented elsewhere in accelerated storage of lipid systems [61,62] and attributable in part to the transition, above approximately 60 °C, from hydroperoxide accumulation to their near-instantaneous decomposition [63].

The attenuation of protection was most evident for AP-E, which lost its significant advantage over CONTROL above 30 °C and even reached a peroxide value at 60 °C (9.94 meq O_2_/kg) numerically higher than the control (9.69 meq O_2_/kg). This is consistent with AP-E being the extract with the lowest phenolic content and highest IC_50_ (Section 3.2.1). Phloridzin, reported as the principal dihydrochalcone of apple pomace, has been described to degrade under comparable thermal conditions [11]. GP-E, in contrast, retained the lowest rate constant at every temperature. This is primarily attributable to its higher phenolic dose (875.8 mg GAE/kg), which provides a larger reserve of active compounds under thermal stress. In addition, heating grape pomace has been reported to release active monomeric phenolics through hydrolytic cleavage of polymeric proanthocyanidins even as more labile compounds are lost [64]. Thermal stress could therefore partially regenerate the pool of active monomers and contribute to the sustained protection of GP-E. As the antioxidant content of the samples was not monitored during storage, the thermal stability of the extracts was not established here, and these interpretations remain hypotheses.

Color change progressed with storage time at the three temperatures and was almost entirely attributable to increasing yellowness, with Δb* accounting for 97.1% of the squared total color difference (ΔE^2^) at day 35. This dominance of the b* coordinate and its direction are consistent with correlations reported between color coordinates and lipid oxidation indices in a repeatedly heated vegetable oil system, where yellowness correlated positively and significantly with PV, p-AV and the total oxidation index, lightness correlated negatively with all of them, and redness showed no significant correlation with either PV or p-AV [65]. The relative contribution of the three coordinates observed therefore reproduces this reported pattern, suggesting that surface color may serve as a complementary descriptor of the progression of lipid oxidation in this matrix. However, initial b* values differed among treatments as a consequence of the intrinsic color of the extracts, an effect documented for lipophilic pigments added to ghee [66,67]; consequently, ΔE may integrate oxidation-related changes together with changes in the pigments contributed by the extracts and was interpreted as a complementary indicator rather than as an independent marker of oxidation.

The direction of the b* change nevertheless requires interpretation, as two opposing processes act on this coordinate in milk fat: the native β-carotene responsible for the golden color of ghee is bleached during oxidation through addition of peroxyl radicals to its conjugated C=C double bonds, reducing yellowness [68], while non-enzymatic browning of residual lactose and free amino groups through Maillard and caramelization reactions generates yellow-to-brown pigments that increase it, together with non-volatile polymers arising from lipid autoxidation [17,68]. These contributions have been described as partially counteracting each other [68], such that the net direction depends on which predominates in a given system.

At 60 °C the change was not confined to yellowness. Lightness, essentially stable at 30 °C (mean ΔL* = −1.26), decreased progressively to −7.24 at 60 °C, concentrated at the final sampling point of the highest temperature, where a* also shifted into the positive quadrant in the least protected samples. This combination of decreasing L*, increasingly positive a* and elevated b* corresponds to the chromatic signature described for non-enzymatic browning in milk fat, attributed to the accumulation of non-volatile decomposition products, Maillard intermediates and high-molecular-weight brown polymers formed in the advanced stages of the reaction [17,67,69]. Its restriction to the highest temperature and to the least protected treatments is consistent with a threshold behavior in which browning becomes chromatically detectable only once primary and secondary oxidation products have accumulated sufficiently.

The behavior of the synthetic blend illustrates that color and chemical indices do not necessarily converge: BHA/BHT reduced total color change by 15.2–23.1%, but without reaching significance against the control at any temperature, despite differing significantly from it in both PV and p-AV. Discrepancies of this kind have been documented in this matrix, with rosemary extract added to ghee maintaining a peroxide value of zero over 210 days at 37 °C and 36 days at 60 °C while organoleptic scores declined progressively, an effect attributed not to lipid autoxidation but to thermal degradation of native volatile compounds and carotenoid pigments [49]; the converse has also been reported, with lycopene added to clarified butter suppressing peroxide formation and free acidity while rendering samples significantly darker [70]. Surface color therefore responds to a broader set of processes than oxidation markers, including pigment degradation and the formation of chromophores [71].

### 4.3. Functionalized Chitosan Films in Active Packaging (Butter Model)

The ranking of the five packaging treatments in delaying lipid oxidation, established across all four indices, mirrors the antioxidant capacity of the corresponding extracts determined in Section 3.2.1, where GP-E showed the lowest IC_50_ and PP-E and AP-E showed comparable, higher IC_50_ values. This correspondence suggests a link between the radical scavenging capacity of the incorporated extract, measured in the free state, and its effectiveness once incorporated into the film and placed in contact with the butter. Comparable results have been reported for chitosan–starch films functionalized with purslane extract, in which the active formulation reduced malondialdehyde accumulation in refrigerated pork by 21.8–28.7% relative to unfunctionalized chitosan, while the unfunctionalized chitosan film in turn outperformed a conventional polyethylene wrap in delaying protein spoilage, although not in curbing lipid oxidation at longer storage times [72].

The fact that the unfunctionalized control film performed worse than the plastic control, rather than merely failing to add protection, is consistent with the reported limitations of chitosan as a packaging material in the absence of an incorporated antioxidant. According to the available literature, unfunctionalized chitosan films possess a comparatively low water vapor barrier owing to the abundance of polar hydroxyl and amino groups in the polymer [2,21], and their oxygen permeability, moderate when dry, increases markedly upon swelling by absorbed moisture in contact with a food surface [73]; chitosan itself possesses negligible intrinsic radical scavenging capacity, lacking readily donatable hydrogen atoms, with DPPH values as low as 2.84–3.81% reported for unfunctionalized films [21,74]. In an anhydrous fat model such as the present one, these reported limitations would be expected to compound, the unfunctionalized film offering neither an effective oxygen barrier nor chemical protection, which is consistent with its failure to improve upon passive packaging control.

Different mechanisms have been proposed for the protection conferred by antioxidant-functionalized packaging: physical or chemical restriction of oxygen transfer [20], migration of the active compound into the food followed by direct radical scavenging within the food matrix [75], and non-migratory interfacial scavenging in which the phenolic compounds remain associated with the film surface [36]. These are not mutually exclusive and are difficult to separate in a real food system, where uncertainty has been noted as to whether the dominant process is migration of the antioxidant into the food or diffusion of oxidizing species from the food into the film [75], with several studies describing them as operating synergistically [36,76]. Migration of phenolic compounds into a food matrix has been documented in the literature, with antioxidant capacity measurable within the food increasing in proportion to extract loading in the film [74], and moisture-driven swelling of the polymer identified as the trigger for this release [20]. The smaller increase in acid value observed for GP-E Film at room temperature (0.19 vs. 1.02 mg KOH/g for the control film) would be consistent with these active mechanisms to the extent that a lower formation of hydroperoxides reduces the short-chain acids released by their decomposition, which are also titrated as acid value; however, as neither extract release nor antioxidant content in the films was quantified, the relative contribution of migration, interfacial scavenging and barrier effects cannot be established from the present data.

The differences among functionalized films cannot be attributed solely to the antioxidant capacity of the extracts. Since the extracts were incorporated on an equal-volume basis, the films carried different phenolic loads, so that their performance reflects both the potency and the dose of each extract. The incorporation of phenolic extracts into chitosan films has been reported to modify their moisture content and water solubility [21,77] and their water vapor permeability, which may decrease through polymer–phenol interactions [21,77] or increase with hygroscopic extracts [72,74]. As these properties were not evaluated, their contribution to the differences observed among films cannot be excluded.

Regarding FT-IR characterization, the displacement of the O–H/N–H stretching band towards lower wavenumbers upon glycerol incorporation in Control-F is consistent with behavior reported for other glycerol-plasticized chitosan systems, with a shift from 3346 to 3309 cm^−1^ described for a biocomposite film [78] and a broad band at 3273 cm^−1^ reported for a chitosan–whey protein film, reflecting the hydrogen-bonding network established between the hydroxyl groups of the plasticizer and the amino groups of chitosan [77]. The mechanism has been described as a two-step process in which glycerol first disrupts the native inter- and intramolecular hydrogen bonds between adjacent chitosan chains and subsequently establishes new bonds between its own hydroxyl groups and the hydroxyl and amino groups of the glucosamine units [79].

The glycosidic C–O–C and pyranose C–O bands remained close to their native positions in Control-F and in the three functionalized films, constituting evidence against covalent modification of the chitosan backbone, since covalent ether coupling has been shown to displace the C–O–C stretching towards higher wavenumbers relative to the unmodified matrix [80], and the stability of this band has accordingly been used as evidence against such a mechanism [81]. No band was detected in the 1255–1275 cm^−1^ interval in chitosan, Control-F or the functionalized films, excluding covalent crosslinking through the quinone–amine pathway, for which the appearance of a new peak at 1258 cm^−1^ assigned to C–N stretching of aromatic amines constitutes the diagnostic evidence [82]; likewise, no band was detected in the 1720–1760 cm^−1^ interval, excluding phenolic ester formation, for which a new peak near 1727 cm^−1^ is the reported criterion [83]. The retention of native skeletal bands alongside the absence of these reaction-specific features supports the non-covalent entrapment of the extracts within the polymer network.

The perturbation of the amide region nevertheless indicates that this entrapment is mediated by specific interactions rather than by mere physical occlusion. Amide I, displaced from 1651 cm^−1^ in chitosan to 1601 cm^−1^ in Control-F, occurred at intermediate positions in the functionalized films, appearing as an unresolved shoulder in AP-F and GP-F and as a discrete band at 1649 cm^−1^ in PP-F; displacement and broadening of this band upon incorporation of phenolic extracts have been attributed to overlap with the C=C and C–C stretching vibrations of aromatic rings of flavonoids and phenolic acids in the 1650–1600 cm^−1^ interval [28,77]. Amide II was displaced to 1555, 1557 and 1560 cm^−1^ in AP-F, PP-F and GP-F respectively, relative to 1558 cm^−1^ in chitosan and in Control-F. Displacements of this band upon incorporation of phenolic extracts have been reported in chitosan nanocapsules loaded with grape pomace polyphenols, being interpreted as evidence of an intermolecular hydrogen-bonding network between the phenolic hydroxyl groups of the extract and the free amino groups or amide carbonyls of chitosan [41].

It is important to notice that the individual phenolic composition of the extracts was not determined; TPC, antioxidant capacity assays and FT-IR provide class-level information only. Therefore, the contribution of specific compounds to the observed behavior, including differences in thermal behavior among extracts, is discussed in light of the currently available literature.

## 5. Conclusions

Cold maceration with absolute ethanol yielded higher TPC and antioxidant capacity than water in the three pomaces, with the largest solvent effect in grape pomace. Extending the extraction time from 24 to 48 h increased both parameters, with statistically significant differences mainly in the ethanolic extracts. Among the selected 48 h ethanolic extracts, GP-E exhibited the highest antioxidant capacity, with an IC_50_ at least 94% lower than AP-E and PP-E in the DPPH and ABTS assays, whereas AP-E and PP-E showed comparable values.

When added to ghee at 0.186% *w*/*w* of dry extract (0.088% *w*/*w* of phenolics, as GAE) under accelerated storage (30–60 °C), GP-E displayed the lowest oxidation rate constants at all temperatures, significantly lower than those of the BHA/BHT blend applied at 0.02% *w*/*w*. PP-E did not differ significantly from the synthetic blend, whereas the protective effect of AP-E decreased with temperature, with no significant difference from the control at 60 °C. The activation energies of BHA/BHT, PP-E and GP-E were significantly higher than the control, whereas the one from AP-E did not differ significantly from it. The treatment ranking in inhibiting surface color change was consistent with the chemical oxidation indices, supporting its use as a complementary descriptor of oxidative deterioration.

In butter packaged with chitosan films and stored for 15 days at 21 and 30 °C, GP-E Film resulted in the lowest oxidation values, below both the unfunctionalized film and the conventional plastic packaging. The oxidation values obtained with PP-E Film did not differ significantly from the plastic packaging. In contrast, AP-E Film showed higher oxidation values than PP-E Film and the plastic packaging, although lower than the unfunctionalized film. These results are consistent with the antioxidant activity of the extracts being retained within the film matrices.

A distinctive aspect of this work is that the same three extracts, obtained under identical conditions, were followed from their characterization through two dairy fat systems, as free additives in ghee with kinetic modeling of oxidation and incorporated into chitosan films for butter. The FT-IR spectra reported in this work also constitute, to our knowledge, the first characterization of a pear pomace phenolic extract.

Overall, these findings provide a basis for the further development of grape and pear pomace extracts as natural antioxidants in both free and confined delivery systems for high-fat dairy matrices.

### Limitations of the Study

Current findings in this work are not without limitations. The individual phenolic composition of the extracts was not determined, and the extracts were incorporated at equal volume rather than at equivalent phenolic concentrations; therefore, the comparisons reflect the performance of each additive at the doses evaluated, and the mechanistic basis of the differential thermal behavior among extracts remains to be established. Since water and ethanol were evaluated only separately, further research should optimize the ethanol–water ratio, establish dose–response relationships at equivalent phenolic concentrations relative to synthetic antioxidants, and complement the spectrophotometric assays (TPC, DPPH, ABTS and FRAP) with the identification and quantification of individual phenolic compounds.

For active packaging applications, determination of the barrier and mechanical properties of the films, quantification of extract release into the food, and sensory evaluation under real storage conditions are needed to support practical implementation.

## Figures and Tables

**Figure 1:**
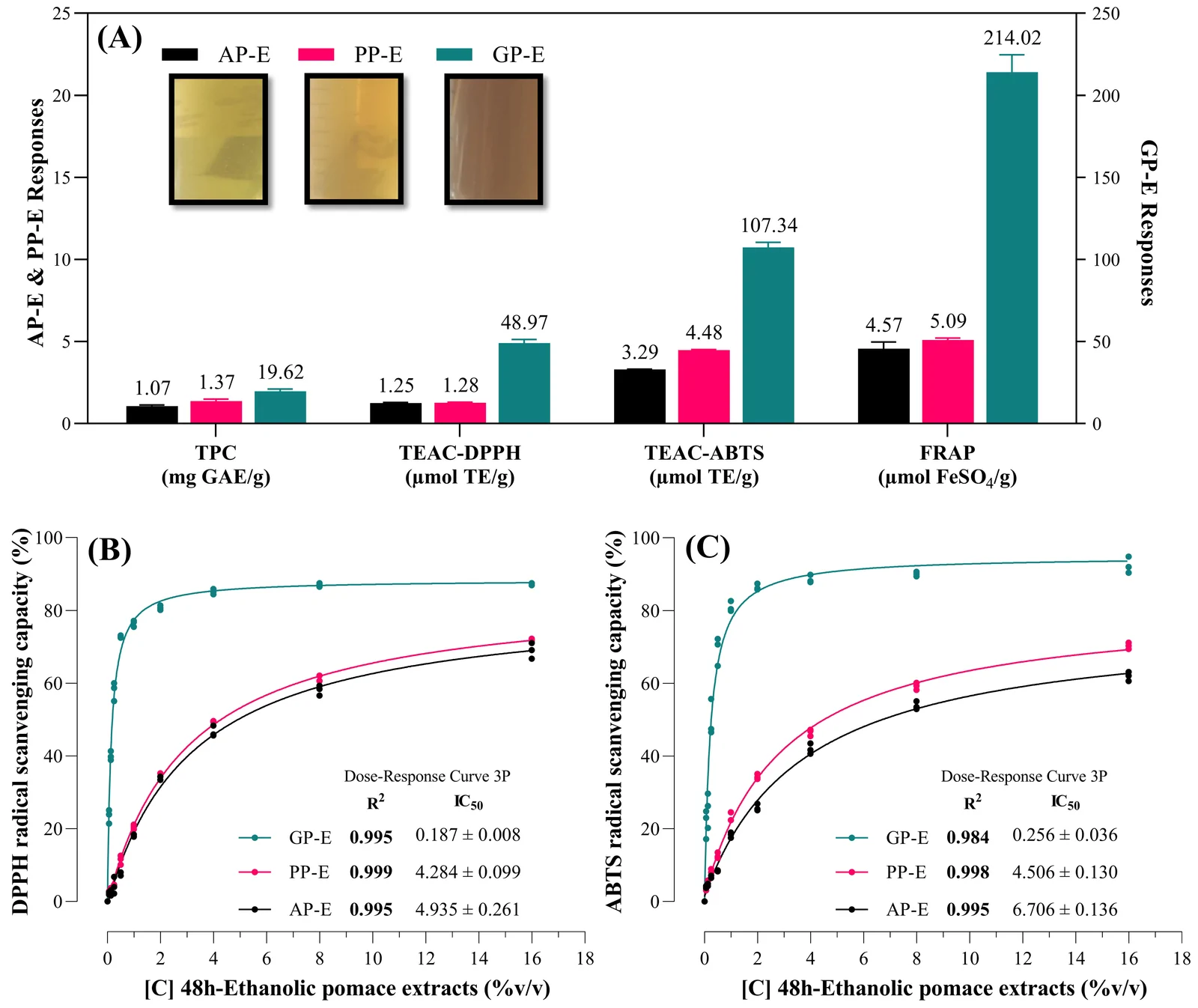
Comparative characterization of 48 h ethanolic pomace extracts from apple (AP-E), pear (PP-E), and grape (GP-E). (**A**) Total phenolic content (TPC) and antioxidant capacity (TEAC-DPPH, TEAC-ABTS, and FRAP) insets show representative photographs of each extract. Note that the y-axis scale differs among extracts, since the values of GP-E are one to two orders of magnitude higher than those of AP-E and PP-E. (**B**) Dose–response curves for DPPH and (**C**) ABTS radical scavenging capacity as a function of extract concentration (%*v*/*v*); lines represent the three-parameter dose–response fitted model (R^2^ ≥ 0.98 for all extracts). Data are presented as mean ± SD (n = 4); IC_50_: half-maximal inhibitory concentration; R^2^: coefficient of determination; TE: Trolox equivalents; GAE: gallic acid equivalents.

**Figure 2:**
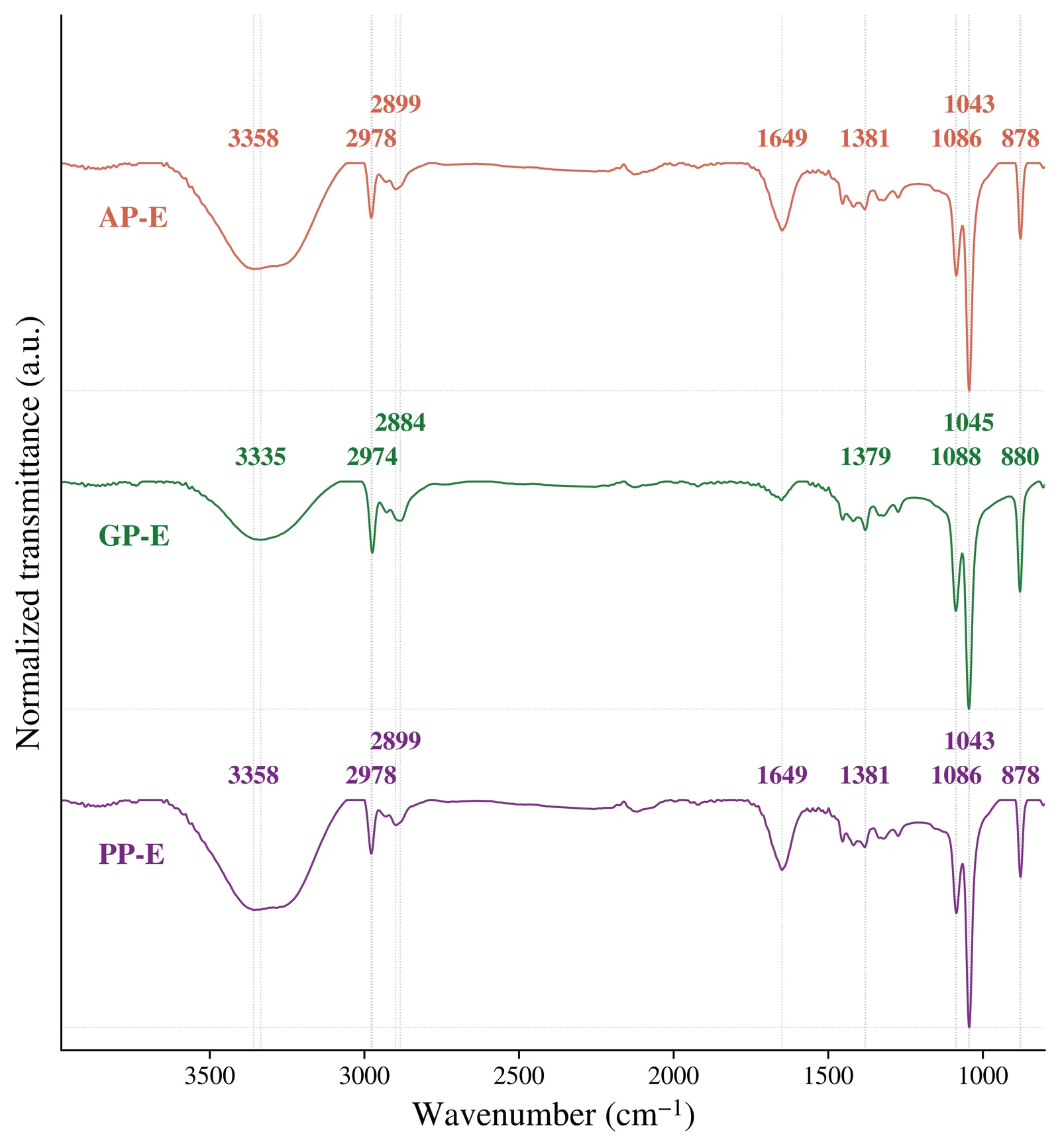
FT-IR spectra of the 48 h ethanolic pomace extracts from apple (AP-E), grape (GP-E), and pear (PP-E), normalized transmittance, in the 4000–650 cm^−1^ region.

**Figure 3:**
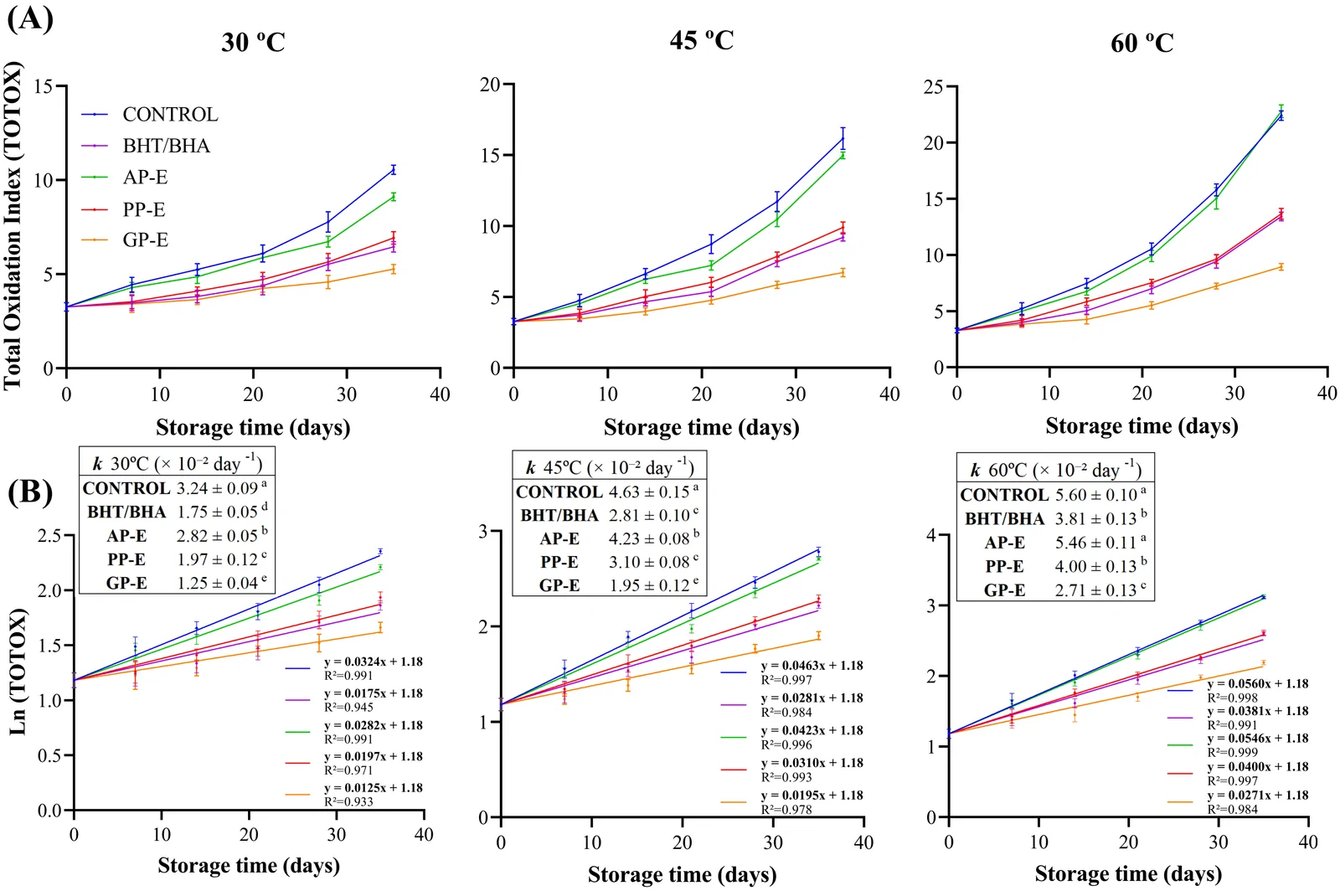
Oxidation kinetics of ghee added with different antioxidant treatments, expressed as the total oxidation index (TOTOX). (**A**) Experimental TOTOX values over 35 days of accelerated storage at 30, 45, or 60 °C. (**B**) Linearized first-order model, shown as in Ln (TOTOX) vs. storage time, with a linear regression fitted to each treatment at each storage temperature (solid lines) constrained to the common initial value, to obtain the corresponding first-order rate constants (*k*, day^−1^), shown as inset tables with different lowercase letters indicating a significant difference among treatments at that temperature (Tukey’s HSD, *p* < 0.05); *k* also differed significantly among the three temperatures within every treatment. Points represent mean ± SD *(n* = 3). CONTROL, ghee without added antioxidant; BHT/BHA, ghee added with a synthetic antioxidant; AP-E, PP-E, and GP-E, ghee added with apple, pear, and grape pomace extracts, respectively.

**Figure 4:**
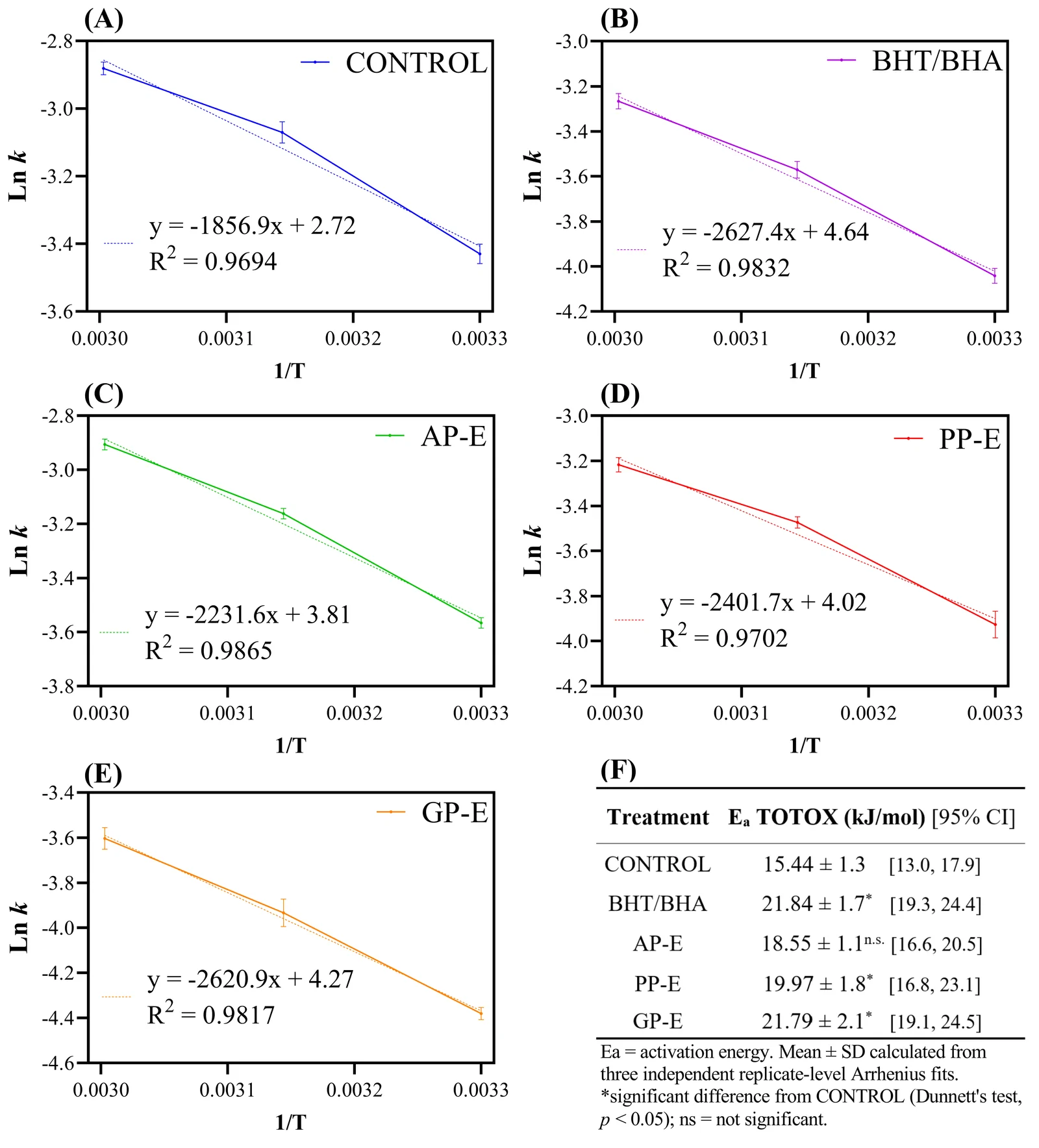
Arrhenius plots of the first-order rate constant (*k*) for TOTOX, shown individually per treatment (**A**–**E**). Points represent mean ± SD of three replicate-level k values. (**F**) Activation energy (Ea) for TOTOX obtained from the slope of each regression, expressed as mean ± SD of three independent replicate-level Arrhenius fits, with the 95% confidence interval (in brackets) calculated from a pooled regression using all nine replicate-level points per treatment. No significant differences were found among the antioxidant-treated groups (Tukey’s HSD).

**Figure 5:**
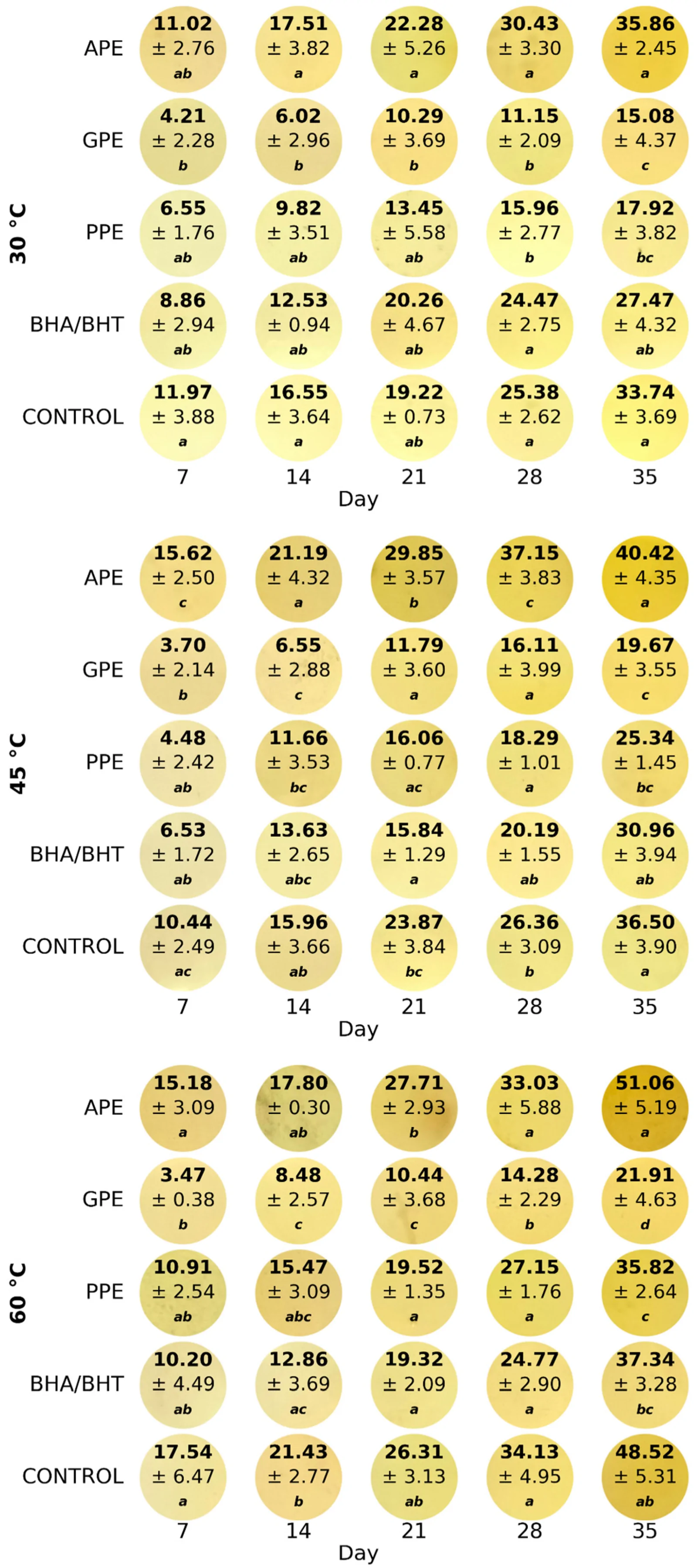
Surface appearance of ghee samples during accelerated storage at 30, 45 and 60 °C. Each circle corresponds to one representative replicate, cropped to a normalized radius; values presented inside each disk are the total color difference (ΔE, mean ± SD, n = 3) relative to its own reading at day 0, taken before storage. Different letters within a column indicate significant differences among treatments at that temperature and sampling day (Tukey HSD, *p* < 0.05); letters are not comparable across columns.

**Figure 6:**
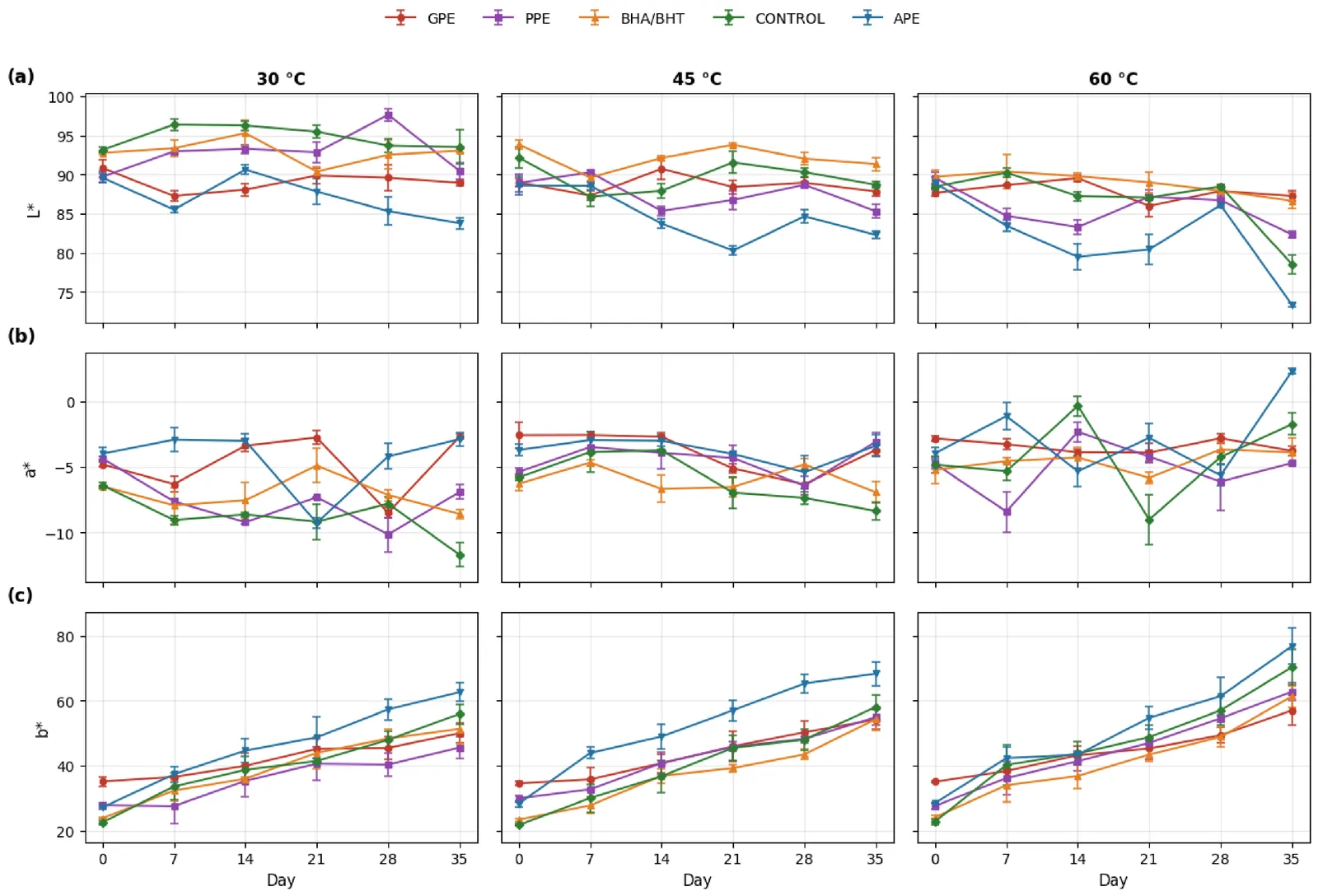
Evolution of the CIELab coordinates of ghee samples during accelerated storage at 30, 45 and 60 °C: (**a**) L*, (**b**) a*, (**c**) b*. Symbols are means of independent replicates (n = 3) and error bars denote the standard deviation.

**Figure 7:**
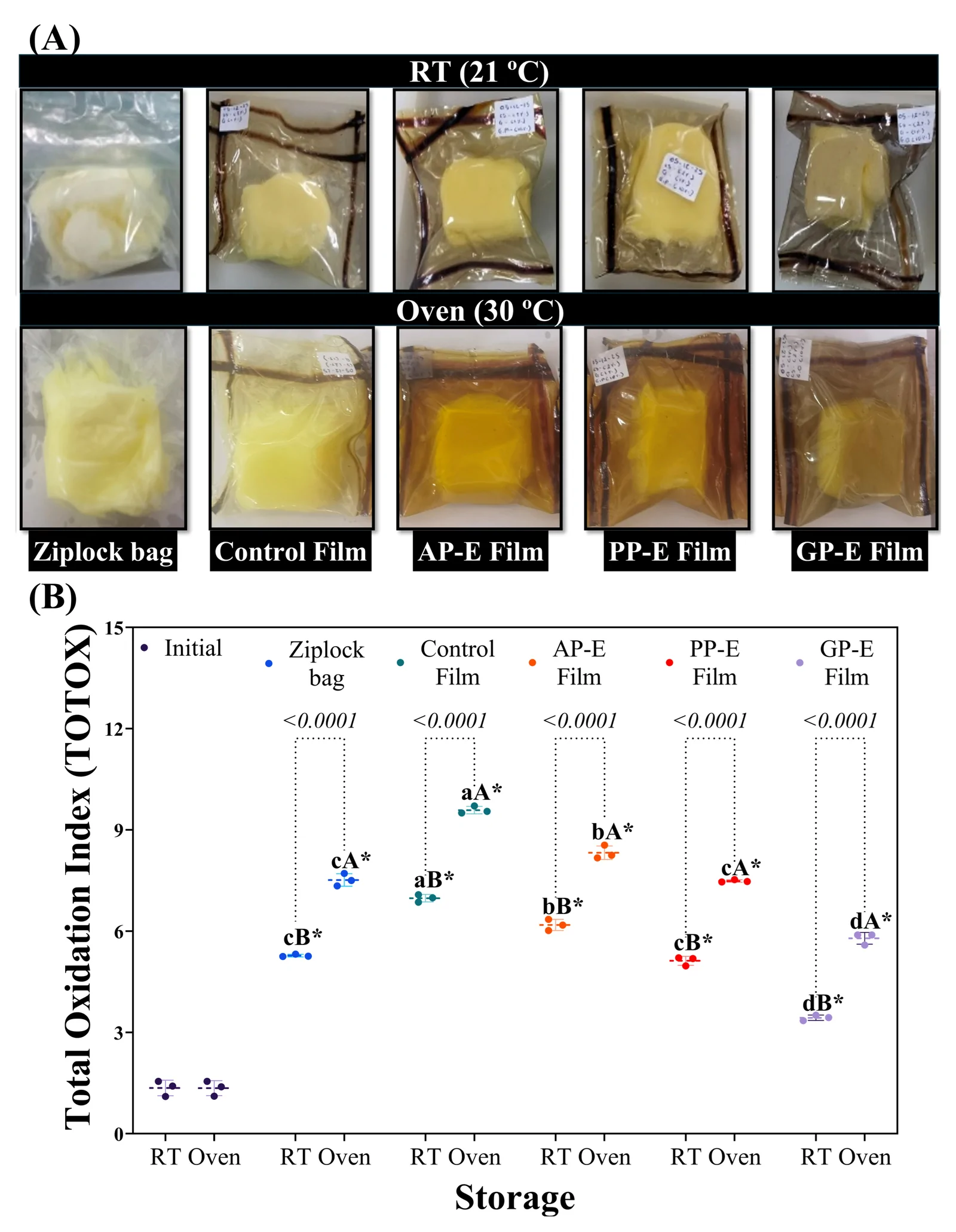
TOTOX values of butter samples wrapped in active chitosan-based films (AP-E Film, PP-E Film, GP-E Film) or kept under reference conditions (Ziplock bag, unfunctionalized control film), compared with the initial (unstored) sample, after 15 days of storage at room temperature (RT, 21 °C) or in an oven (30 °C). (**A**) Appearance of the butter samples after storage under each packaging treatment. (**B**) TOTOX values; symbols represent individual observations (*n* = 3), and horizontal lines with error bars indicate the mean ± SD. A two-way ANOVA (Treatment × Storage condition) was performed. Lowercase letters compare treatments within the same storage condition, and uppercase letters compare RT and oven within the same treatment (Tukey’s HSD, *p* < 0.05). Asterisks indicate a significant difference from the initial (day 0) value (Dunnett’s test, *p* < 0.05).

**Figure 8:**
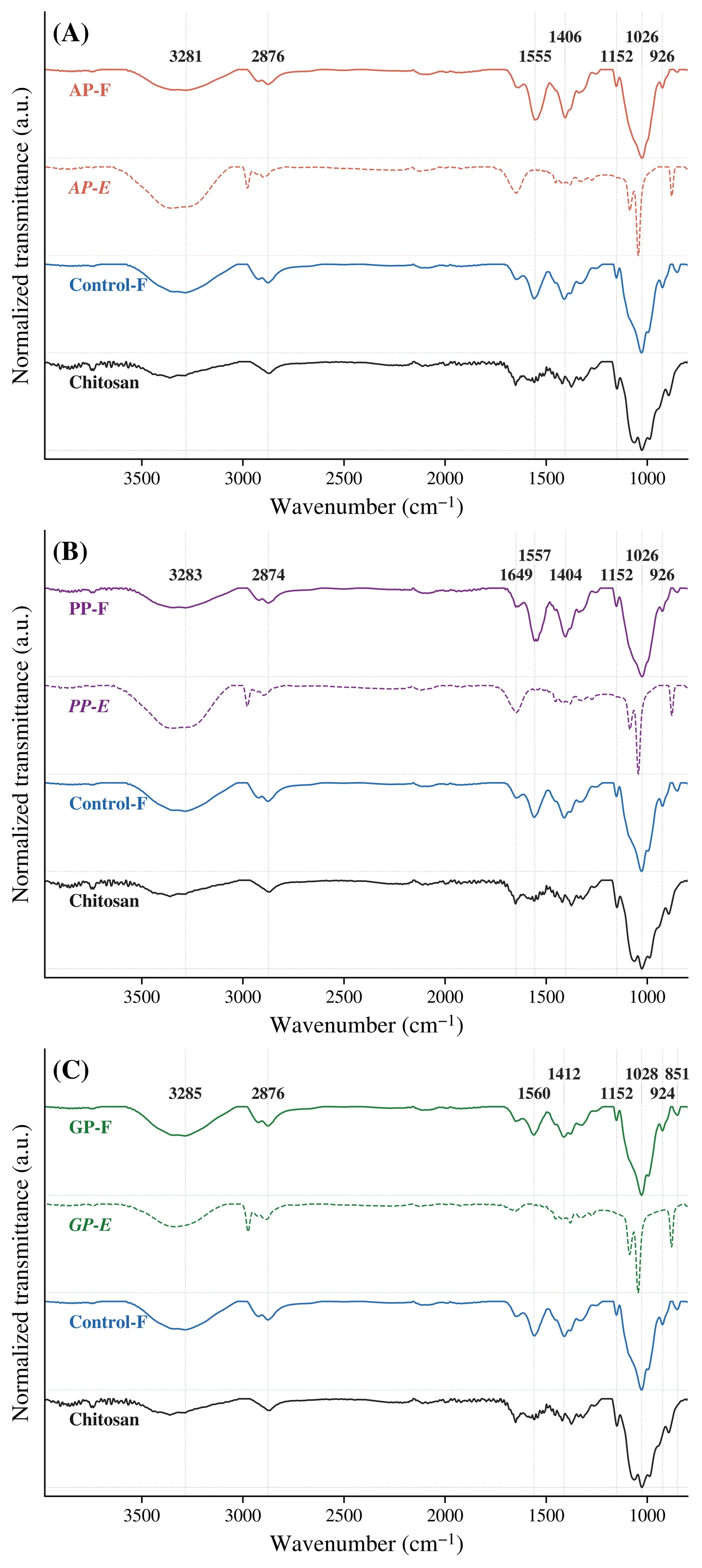
Comparative FT-IR spectra of the functionalized chitosan-based films (AP-Film, PP-Film, GP-Film), the corresponding pomace extract used for functionalization (AP-E, PP-E, GP-E), the unfortified control film (Control-F), and neat chitosan, in the 4000–650 cm^−1^ region. (**A**) Apple system (AP-Film, AP-E); (**B**) pear system (PP-Film, PP-E); (**C**) grape system (GP-Film, GP-E).

**Table 1:** Full factorial design (2^2^) for pomace extraction by cold maceration.

Treatment	Solvent	Extraction Time (h)
T1	Distilled water	24
T2	Distilled water	48
T3	Absolute ethanol (≥99.5%)	24
T4	Absolute ethanol (≥99.5%)	48

**Table 2:** Experimental treatments evaluated in ghee.

Code	Treatment	Description
	Natural antioxidants	* Dry equivalents to 10% *v*/*v*
AP-E	Apple pomace extract	914.1 (mg extract/kg ghee)
PP-E	Pear pomace extract	937.5 (mg extract/kg ghee)
GP-E	Grape pomace extract	1856.0 (mg extract/kg ghee)
CONTROL	Vehicle control	Ghee without antioxidants
BHA/BHT	Synthetic antioxidants	(1:1 *w*/*w*) at 200 (mg/kg ghee)

* Extracts were applied on an equal-volume basis.

**Table 3:** Total phenolic content (TPC) and antioxidant capacity (DPPH, ABTS, and FRAP) of apple, pear, and grape pomace extracts obtained by cold maceration with distilled water or absolute ethanol for 24 or 48 h.

Pomace Source	Extraction Time (h)	Water	Ethanol
TPC (mg GAE/g)			
Apple	24	0.65 ± 0.10 ^aA^	0.99 ± 0.09 ^bA^
	48	0.76 ± 0.06 ^aA^	1.07 ± 0.07 ^bA^
Pear	24	0.61 ± 0.03 ^aA^	1.06 ± 0.06 ^bA^
	48	0.67 ± 0.05 ^aA^	1.37 ± 0.12 ^bB^
Grape	24	7.02 ± 0.24 ^aA^	9.53 ± 0.52 ^bA^
	48	9.95 ± 0.36 ^aB^	19.62 ± 1.45 ^bB^
TEAC-DPPH (µmol TE/g)			
Apple	24	0.27 ± 0.02 ^aA^	1.09 ± 0.04 ^bA^
	48	0.36 ± 0.02 ^aB^	1.25 ± 0.04 ^bB^
Pear	24	0.80 ± 0.06 ^aA^	1.19 ± 0.03 ^bA^
	48	0.97 ± 0.02 ^aB^	1.28 ± 0.03 ^bB^
Grape	24	4.25 ± 0.22 ^aA^	20.39 ± 1.13 ^bA^
	48	5.95 ± 0.45 ^aA^	48.97 ± 2.33 ^bB^
TEAC-ABTS (µmol TE/g)			
Apple	24	1.74 ± 0.07 ^aA^	2.93 ± 0.08 ^bA^
	48	2.08 ± 0.05 ^aB^	3.29 ± 0.03 ^bB^
Pear	24	1.84 ± 0.04 ^aA^	3.66 ± 0.05 ^bA^
	48	2.32 ± 0.01 ^aB^	4.48 ± 0.04 ^bB^
Grape	24	7.11 ± 1.21 ^aA^	50.64 ± 2.30 ^bA^
	48	10.09 ± 1.71 ^aA^	107.34 ± 3.07 ^bB^
FRAP (µmol FeSO_4_/g)			
Apple	24	1.35 ± 0.12 ^aA^	4.62 ± 0.15 ^bA^
	48	1.45 ± 0.10 ^aA^	5.09 ± 0.12 ^bB^
Pear	24	1.44 ± 0.08 ^aA^	3.72 ± 0.05 ^bA^
	48	1.85 ± 0.16 ^aA^	4.57 ± 0.40 ^bB^
Grape	24	42.24 ± 0.81 ^aA^	106.69 ± 5.26 ^bA^
	48	45.70 ± 1.80 ^aA^	214.02 ± 10.61 ^bB^

Data are expressed as mean ± SD (n = 4). For each pomace source and parameter, an independent two-way ANOVA (Solvent × Time) was performed. Different lowercase letters (a, b) within the same row indicate significant differences (Tukey’s HSD, *p* < 0.05) between solvents at a given extraction time; different uppercase letters (A, B) within the same column indicate significant differences between extraction times for a given solvent. Letters are only comparable within the same pomace source and parameter block. GAE: gallic acid equivalents; TE: Trolox equivalents.

**Table 4:** Comparison of total phenolic content (TPC) and antioxidant capacity (DPPH, ABTS, and FRAP) among apple, pear, and grape pomace extracts (marginal means, pooled across solvent and extraction time). Welch’s ANOVA and Games–Howell post hoc test.

Pomace Source	Mean ± SD	Grouping	Welch’s F (df1, df2)	*p*-Value
	TPC (mg GAE/g)			
Apple	0.87 ± 0.19	b	35.32 (2, 24.6)	<0.001
Pear	0.93 ± 0.33	b		
Grape	11.53 ± 5.01	a		
	TEAC-DPPH (µmol TE/g)			
Apple	0.74 ± 0.45	c	11.40 (2, 23.2)	<0.001
Pear	1.06 ± 0.20	b		
Grape	19.89 ± 18.55	a		
	TEAC-ABTS (µmol TE/g)			
Apple	2.51 ± 0.65	b	9.15 (2, 24.8)	0.001
Pear	3.07 ± 1.08	b		
Grape	43.79 ± 41.89	a		
	FRAP (µmol FeSO_4_/g)			
Apple	3.13 ± 1.80	b	14.90 (2, 26.0)	<0.001
Pear	2.89 ± 1.35	b		
Grape	102.16 ± 71.96	a		

Data are expressed as mean ± SD, pooled across the two solvents and two extraction times (n = 16 per pomace source). Welch’s ANOVA was used instead of the classical F-test because Levene’s test indicated unequal variances among pomace sources; Games–Howell post hoc test was applied accordingly. df1 and df2 are the numerator and denominator (error) degrees of freedom, respectively. Different letters within the same parameter indicate significant differences among pomace sources (*p* < 0.05). GAE: gallic acid equivalents; TE: Trolox equivalents.

**Table 5:** Acid value (AV), peroxide value (PV), and p-anisidine value (p-AV) of ghee samples after 35 days of accelerated storage at 30, 45, or 60 °C.

Treatment	Storage Temperature		
	30 °C	45 °C	60 °C
AV (mg KOH/g)			
Initial	0.42 ± 0.02		
CONTROL	1.44 ± 0.03 ^aC*^	1.96 ± 0.09 ^aB*^	2.68 ± 0.03 ^aA*^
BHT/BHA	0.94 ± 0.06 ^cC*^	1.25 ± 0.03 ^bB*^	1.75 ± 0.07 ^bA*^
AP-E	1.24 ± 0.04 ^bC*^	1.84 ± 0.04 ^aB*^	2.68 ± 0.08 ^aA*^
PP-E	0.97 ± 0.06 ^cC*^	1.32 ± 0.03 ^bB*^	1.74 ± 0.07 ^bA*^
GP-E	0.78 ± 0.03 ^dC*^	0.94 ± 0.03 ^cB*^	1.25 ± 0.05 ^cA*^
PV (meq O_2_/kg)			
Initial	1.36 ± 0.10		
CONTROL	4.28 ± 0.15 ^aC*^	6.95 ± 0.34 ^aB*^	9.69 ± 0.24 ^aA*^
BHT/BHA	2.52 ± 0.09 ^cC*^	3.75 ± 0.19 ^bB*^	5.59 ± 0.15 ^bA*^
AP-E	3.71 ± 0.14 ^bC*^	6.40 ± 0.17 ^aB*^	9.94 ± 0.23 ^aA*^
PP-E	2.77 ± 0.12 ^cC*^	4.07 ± 0.21 ^bB*^	5.74 ± 0.20 ^bA*^
GP-E	2.04 ± 0.14 ^dC*^	2.68 ± 0.15 ^cB*^	3.56 ± 0.11 ^cA*^
p-AV (dimensionless)			
Initial	0.54 ± 0.03		
CONTROL	1.98 ± 0.11 ^aC*^	2.26 ± 0.13 ^aB*^	3.01 ± 0.09 ^aA*^
BHT/BHA	1.42 ± 0.12 ^bB*^	1.70 ± 0.13 ^bB*^	2.26 ± 0.15 ^bA*^
AP-E	1.71 ± 0.12 ^aC*^	2.18 ± 0.16 ^aB*^	2.90 ± 0.12 ^aA*^
PP-E	1.40 ± 0.12 ^bC*^	1.77 ± 0.04 ^bB*^	2.18 ± 0.11 ^bA*^
GP-E	1.20 ± 0.08 ^bB*^	1.35 ± 0.04 ^cB*^	1.79 ± 0.10 ^cA*^

Data are expressed as mean ± SD (n = 3). For each indicator, a two-way ANOVA (Treatment × Temperature) was performed on the day-35 data; lowercase letters compare treatments within the same temperature and uppercase letters compare temperatures within the same treatment (Tukey’s HSD, *p* < 0.05). Asterisks indicate a significant difference from the initial (day-0 baseline) value (Dunnett’s test, *p* < 0.05).

**Table 6:** Acid value (AV), peroxide value (PV), and p-anisidine value (p-AV) of butter samples stored at room temperature (RT, 21 °C) or under accelerated oxidation conditions (oven, 30 °C).

Treatment	AV (mg KOH/g)		PV (meq O_2_/kg)		p-AV (Dimensionless)	
Storage	RT	Oven	RT	Oven	RT	Oven
Initial	0.60 ± 0.10		0.27 ± 0.08		0.81 ± 0.10	
Ziplock bag	1.06 ± 0.06 ^cB^*	1.62 ± 0.11 ^cA^*	1.17 ± 0.06 ^cB^*	1.65 ± 0.05 ^cA^*	2.93 ± 0.12 ^cB^*	4.22 ± 0.11 ^cA^*
Control Film	1.62 ± 0.08 ^aB^*	2.51 ± 0.10 ^aA^*	1.76 ± 0.09 ^aB^*	2.21 ± 0.06 ^aA^*	3.46 ± 0.09 ^aB^*	5.17 ± 0.08 ^aA^*
AP-E Film	1.39 ± 0.09 ^bB^*	1.85 ± 0.05 ^bA^*	1.50 ± 0.11 ^bB^*	1.87 ± 0.06 ^bA^*	3.18 ± 0.08 ^bB^*	4.58 ± 0.10 ^bA^*
PP-E Film	1.20 ± 0.10 ^bB^*	1.71 ± 0.03 ^bA^*	1.12 ± 0.03 ^cB^*	1.69 ± 0.07 ^cA^*	2.89 ± 0.11 ^cB^*	4.11 ± 0.09 ^cA^*
GP-E Film	0.79 ± 0.08 ^dB^	1.20 ± 0.10 ^dA^*	0.59 ± 0.06 ^dB^*	1.23 ± 0.07 ^dA^*	2.26 ± 0.05 ^dB^*	3.32 ± 0.04 ^dA^*

Data are expressed as mean ± SD (*n* = 3). For each indicator, a two-way ANOVA (Treatment × Storage condition) was performed; lowercase letters compare treatments within the same column and uppercase letters compare RT and oven within the same treatment and parameter (Tukey’s HSD, *p* < 0.05). Asterisks indicate a significant difference from the initial (day-0 baseline) value (Dunnett’s test, *p* < 0.05).

## Data Availability

The original contributions presented in this study are included in the article. Further inquiries can be directed to the corresponding author.

## Supplementary Materials

The following supporting information can be downloaded at https://www.mdpi.com/article/10.3390/foods15203736/s1, Figure S1: Calibration curves used for the quantification of total phenolic content and antioxidant capacity of pomace extracts; Table S1: Complete extract characterization data (TPC, TEAC-DPPH, TEAC-ABTS, and FRAP) for apple, pear, and grape pomace extracts obtained at two extraction times (24 or 48 h) using ethanol or water as solvent, with individual replicates and summary statistics; Table S2: Complete AV data (acid value) for all treatments, temperatures, and storage days, with individual replicates and summary statistics; Table S3: Complete PV data (meq O2/kg) for all treatments, temperatures, and storage days, with individual replicates and summary statistics; Table S4: Complete p-AV data for all treatments, temperatures, and storage days, with individual replicates and summary statistics; Table S5: Complete TOTOX data for all treatments, temperatures, and storage days, with individual replicates and summary statistics; Table S6: Complete AV, PV, p-AV, and TOTOX data for butter samples stored under each film treatment at room temperature (RT, 21 °C) or in an oven (oven, 30 °C), with individual replicates and summary statistics.

## Abbreviations

The following abbreviations are used in this manuscript:

ABTS	2,2-azino-bis(3-ethylbenzothiazoline-6-sulfonate)
ANOVA	Analysis of variance
AOCS	American Oil Chemists’ Society
AP-E	Apple pomace extract
AP-F	Chitosan film functionalized with apple pomace extract
ASLT	Accelerated shelf-life testing
ATR	Attenuated total reflectance
AV	Acid value
BHA	Butylated hydroxyanisole
BHT	Butylated hydroxytoluene
CIELab	CIE L*a*b* color space
Control-F	Unfunctionalized chitosan control film
DPPH	1,1-diphenyl-2-picrylhydrazyl
E_a_	Activation energy
FRAP	Ferric reducing antioxidant power
FT-IR	Fourier transform infrared spectroscopy
GAEs	Gallic acid equivalents
GP-E	Grape pomace extract
GP-F	Chitosan film functionalized with grape pomace extract
HSD	Honestly significant difference
IC_50_	Half-maximal inhibitory concentration
k	Apparent first-order rate constant
p-AV	p-Anisidine value
PP-E	Pear pomace extract
PP-F	Chitosan film functionalized with pear pomace extract
PV	Peroxide value
RMSE	Root mean square error
RT	Room temperature
SD	Standard deviation
TEs	Trolox equivalents
TEAC	Trolox equivalent antioxidant capacity
TOTOX	Total oxidation index
TPC	Total phenolic content
ΔE	Total color difference
