# Supplementary material for: Application of Antioxidants from Agro-Industrial By-Products in Dairy Fat Systems: Oxidation Kinetics of Ghee and Active Packaging for Butter
Source: Foods. 2026 Oct 20;15(20):3736. doi: 10.3390/foods15203736 (PMC13704126; doi:10.3390/foods15203736)
Supplement: Supplementary file 1 [file foods-15-03736-s001.zip › foods-4598829-supplementary.pdf]

Application of Antioxidants from Agro-Industrial By-products in Dairy Fat Systems: Oxidation Kinetics of Ghee and Active Packaging for Butter

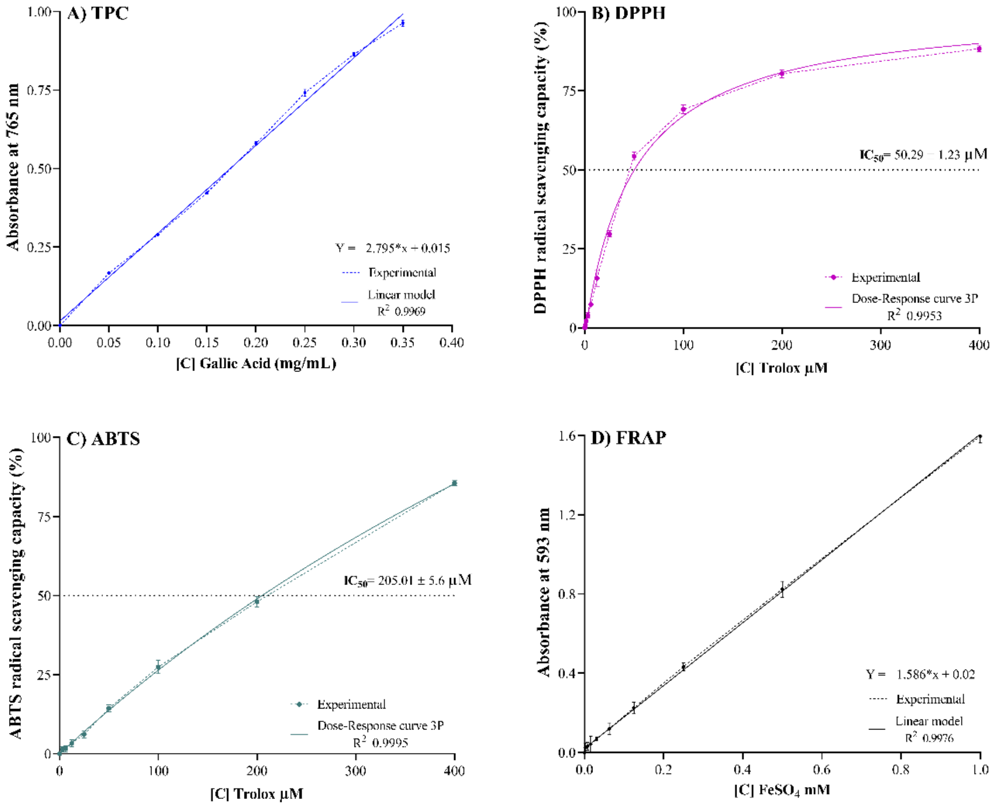

Figure S1. Calibration curves used for the quantification of total phenolic content and antioxidant capacity of pomace extracts. (A) Total phenolic content (TPC), determined by the Folin-Ciocalteu assay using gallic acid as standard; (B) DPPH radical scavenging capacity, using Trolox as standard, fitted to a three-parameter dose-response model; (C) ABTS radical scavenging capacity, using Trolox as standard, fitted to a three-parameter dose-response model; (D) Ferric Reducing Antioxidant Power (FRAP), using $FeSO_4$ as standard. Data points represent the mean ± standard deviation (SD) of the calibration replicates, dotted lines represent the experimental data, and solid lines represent the fitted model. R^2: coefficient of determination.

The following tables report the individual replicates of the results analyzed in the full factorial design for extraction conditions selection (**Table S1**), in the ghee samples during 35 days of accelerated storage at 30, 45, or 60 °C (**Tables S2-S5**) and in the butter samples stored under each film treatment at room temperature (21 °C) or in an oven at 30 °C (**Table S6**).

Full factorial design data: Pomace extracts

Table S1. Complete extract characterization data (TPC, TEAC-DPPH, TEAC-ABTS, and FRAP) for apple, pear, and grape pomace extracts obtained at two extraction times (24 or 48 h) using ethanol or water as solvent, with individual replicates and summary statistics.

Parameter	Source	Solvent	Time	Rep 1	Rep 2	Rep 3	Rep 4	Mean	SD
ABTS	Apple	EtOH	24h	2.88	3.01	2.86	2.99	2.94	0.08
ABTS	Apple	EtOH	48h	3.26	3.30	3.33	3.28	3.29	0.03
ABTS	Apple	Water	24h	1.65	1.81	1.76	1.76	1.75	0.07
ABTS	Apple	Water	48h	2.11	2.00	2.11	2.09	2.08	0.05
ABTS	Grape	EtOH	24h	49.14	50.29	54.00	49.14	50.64	2.30
ABTS	Grape	EtOH	48h	109.67	103.19	106.83	109.67	107.34	3.07
ABTS	Grape	Water	24h	8.32	5.80	6.38	7.93	7.11	1.21
ABTS	Grape	Water	48h	10.48	9.89	7.93	12.06	10.09	1.71
ABTS	Pear	EtOH	24h	3.67	3.71	3.65	3.60	3.66	0.05
ABTS	Pear	EtOH	48h	4.47	4.44	4.47	4.54	4.48	0.04
ABTS	Pear	Water	24h	1.81	1.82	1.89	1.85	1.84	0.04
ABTS	Pear	Water	48h	2.33	2.31	2.31	2.32	2.32	0.01
DPPH	Apple	EtOH	24h	1.09	1.13	1.10	1.03	1.09	0.04
DPPH	Apple	EtOH	48h	1.27	1.28	1.19	1.27	1.25	0.04
DPPH	Apple	Water	24h	0.25	0.27	0.28	0.30	0.28	0.02
DPPH	Apple	Water	48h	0.38	0.36	0.34	0.37	0.36	0.02
DPPH	Grape	EtOH	24h	18.70	21.12	20.87	20.87	20.39	1.13
DPPH	Grape	EtOH	48h	46.96	50.99	46.96	50.99	48.98	2.33
DPPH	Grape	Water	24h	4.24	4.01	4.24	4.54	4.26	0.22
DPPH	Grape	Water	48h	6.25	6.25	6.02	5.30	5.96	0.45
DPPH	Pear	EtOH	24h	1.15	1.21	1.21	1.19	1.19	0.03
DPPH	Pear	EtOH	48h	1.24	1.28	1.28	1.30	1.27	0.03
DPPH	Pear	Water	24h	0.86	0.77	0.84	0.72	0.80	0.06

DPPH	Pear	Water	48h	0.96	0.96	0.99	0.95	0.96	0.02
FRAP	Apple	EtOH	24h	4.47	4.58	4.61	4.83	4.62	0.15
FRAP	Apple	EtOH	48h	5.00	5.02	5.07	5.27	5.09	0.12
FRAP	Apple	Water	24h	1.17	1.35	1.46	1.40	1.34	0.13
FRAP	Apple	Water	48h	1.36	1.50	1.37	1.56	1.45	0.10
FRAP	Grape	EtOH	24h	114.10	101.81	106.22	104.65	106.69	5.26
FRAP	Grape	EtOH	48h	208.35	203.93	215.60	228.20	214.02	10.61
FRAP	Grape	Water	24h	42.55	41.29	41.92	43.18	42.23	0.81
FRAP	Grape	Water	48h	47.28	46.65	43.18	45.70	45.70	1.80
FRAP	Pear	EtOH	24h	3.68	3.66	3.74	3.78	3.71	0.06
FRAP	Pear	EtOH	48h	4.72	4.22	4.26	5.07	4.57	0.40
FRAP	Pear	Water	24h	1.36	1.37	1.49	1.53	1.44	0.09
FRAP	Pear	Water	48h	1.65	2.06	1.86	1.84	1.85	0.17
TPC	Apple	EtOH	24h	0.86	0.99	1.07	1.03	0.99	0.09
TPC	Apple	EtOH	48h	1.00	1.10	1.01	1.15	1.06	0.07
TPC	Apple	Water	24h	0.72	0.60	0.76	0.54	0.66	0.10
TPC	Apple	Water	48h	0.68	0.78	0.78	0.81	0.76	0.06
TPC	Grape	EtOH	24h	10.24	9.29	9.05	9.53	9.53	0.51
TPC	Grape	EtOH	48h	17.89	20.27	19.08	21.23	19.62	1.45
TPC	Grape	Water	24h	6.90	6.90	7.38	6.90	7.02	0.24
TPC	Grape	Water	48h	10.24	9.77	9.53	10.24	9.95	0.35
TPC	Pear	EtOH	24h	1.00	1.01	1.10	1.13	1.06	0.06
TPC	Pear	EtOH	48h	1.22	1.53	1.38	1.36	1.37	0.13
TPC	Pear	Water	24h	0.63	0.58	0.59	0.64	0.61	0.03
TPC	Pear	Water	48h	0.70	0.65	0.72	0.61	0.67	0.05

Values are individual replicates (Rep 1-4), followed by mean ± SD (n = 4), for each Parameter × Source × Solvent × Time combination. TPC is expressed as mg GAE/g, TEAC-DPPH and TEAC-ABTS as μmol TE/g, and FRAP as μmol $FeSO_4$/g.

Oxidation Kinetics in Ghee (AV, PV, p-AV, TOTOX)

Table S2. Complete AV data (acid value) for all treatments, temperatures, and storage days, with individual replicates and summary statistics.

Day	Temp (°C)	Treatment	Rep 1	Rep 2	Rep 3	Mean	SD
7	30	APE	0.56	0.49	0.58	0.54	0.05
7	30	BHT/BHA	0.48	0.44	0.41	0.44	0.04
7	30	CONTROL	0.56	0.65	0.54	0.58	0.06
7	30	GPE	0.48	0.38	0.43	0.43	0.05
7	30	PPE	0.50	0.46	0.40	0.45	0.05
7	45	APE	0.60	0.57	0.61	0.59	0.02
7	45	BHT/BHA	0.54	0.44	0.52	0.50	0.05
7	45	CONTROL	0.59	0.70	0.65	0.65	0.06
7	45	GPE	0.48	0.44	0.46	0.46	0.02
7	45	PPE	0.45	0.54	0.52	0.50	0.05
7	60	APE	0.70	0.66	0.65	0.67	0.03
7	60	BHT/BHA	0.61	0.55	0.50	0.55	0.06
7	60	CONTROL	0.69	0.78	0.65	0.71	0.07
7	60	GPE	0.47	0.53	0.53	0.51	0.03
7	60	PPE	0.62	0.54	0.52	0.56	0.05
14	30	APE	0.59	0.58	0.68	0.62	0.06
14	30	BHT/BHA	0.50	0.53	0.44	0.49	0.05
14	30	CONTROL	0.69	0.76	0.65	0.70	0.06
14	30	GPE	0.50	0.45	0.46	0.47	0.03
14	30	PPE	0.56	0.48	0.54	0.53	0.04
14	45	APE	0.81	0.70	0.76	0.76	0.06
14	45	BHT/BHA	0.61	0.53	0.63	0.59	0.05
14	45	CONTROL	0.86	0.84	0.81	0.84	0.03
14	45	GPE	0.53	0.56	0.47	0.52	0.05
14	45	PPE	0.57	0.70	0.56	0.61	0.08
14	60	APE	0.92	0.86	0.91	0.90	0.03
14	60	BHT/BHA	0.64	0.66	0.65	0.65	0.01
14	60	CONTROL	1.03	0.98	0.95	0.99	0.04

Day	Temp (°C)	Treatment	Rep 1	Rep 2	Rep 3	Mean	SD
14	60	GPE	0.56	0.59	0.55	0.57	0.02
14	60	PPE	0.74	0.80	0.68	0.74	0.06
21	30	APE	0.73	0.70	0.73	0.72	0.02
21	30	BHT/BHA	0.54	0.51	0.65	0.57	0.07
21	30	CONTROL	0.77	0.80	0.90	0.82	0.07
21	30	GPE	0.54	0.51	0.54	0.53	0.02
21	30	PPE	0.57	0.56	0.67	0.60	0.06
21	45	APE	0.87	0.83	0.94	0.88	0.06
21	45	BHT/BHA	0.66	0.73	0.70	0.70	0.04
21	45	CONTROL	1.06	1.02	1.08	1.05	0.03
21	45	GPE	0.58	0.62	0.56	0.59	0.03
21	45	PPE	0.71	0.75	0.78	0.75	0.04
21	60	APE	1.28	1.32	1.19	1.26	0.07
21	60	BHT/BHA	0.94	0.92	0.88	0.91	0.03
21	60	CONTROL	1.40	1.36	1.31	1.36	0.05
21	60	GPE	0.68	0.73	0.77	0.73	0.05
21	60	PPE	0.96	0.96	0.98	0.97	0.01
28	30	APE	0.89	0.93	0.87	0.90	0.03
28	30	BHT/BHA	0.70	0.72	0.80	0.74	0.05
28	30	CONTROL	1.00	1.03	1.12	1.05	0.06
28	30	GPE	0.57	0.59	0.69	0.62	0.06
28	30	PPE	0.81	0.70	0.75	0.75	0.06
28	45	APE	1.24	1.25	1.37	1.29	0.07
28	45	BHT/BHA	0.98	0.91	1.00	0.96	0.05
28	45	CONTROL	1.41	1.40	1.46	1.42	0.03
28	45	GPE	0.78	0.78	0.73	0.76	0.03
28	45	PPE	1.00	0.99	0.99	0.99	0.01
28	60	APE	1.81	1.95	1.76	1.84	0.10
28	60	BHT/BHA	1.17	1.35	1.27	1.26	0.09
28	60	CONTROL	1.98	1.92	1.89	1.93	0.05
28	60	GPE	0.97	0.98	0.98	0.98	0.01

Day	Temp (°C)	Treatment	Rep 1	Rep 2	Rep 3	Mean	SD
28	60	PPE	1.28	1.29	1.21	1.26	0.04
35	30	APE	1.25	1.20	1.27	1.24	0.04
35	30	BHT/BHA	1.01	0.92	0.90	0.94	0.06
35	30	CONTROL	1.46	1.45	1.40	1.44	0.03
35	30	GPE	0.76	0.81	0.78	0.78	0.03
35	30	PPE	0.90	1.02	1.00	0.97	0.06
35	45	APE	1.83	1.89	1.81	1.84	0.04
35	45	BHT/BHA	1.24	1.28	1.22	1.25	0.03
35	45	CONTROL	1.98	1.86	2.04	1.96	0.09
35	45	GPE	0.97	0.92	0.94	0.94	0.03
35	45	PPE	1.35	1.30	1.31	1.32	0.03
35	60	APE	2.59	2.73	2.71	2.68	0.08
35	60	BHT/BHA	1.67	1.80	1.78	1.75	0.07
35	60	CONTROL	2.70	2.69	2.65	2.68	0.03
35	60	GPE	1.21	1.30	1.25	1.25	0.05
35	60	PPE	1.82	1.72	1.69	1.74	0.07

Values are individual replicates (Rep 1–3), followed by mean ± SD (n = 3), for each Treatment × Temperature × Day combination.

Table S3. Complete PV data (meq O2/kg) for all treatments, temperatures, and storage days, with individual replicates and summary statistics.

Day	Temp (°C)	Treatment	Rep 1	Rep 2	Rep 3	Mean	SD
7	30	APE	1.91	1.69	1.80	1.80	0.11
7	30	BHT/BHA	1.63	1.35	1.34	1.44	0.16
7	30	CONTROL	1.75	2.01	1.76	1.84	0.15
7	30	GPE	1.67	1.30	1.36	1.44	0.20
7	30	PPE	1.65	1.46	1.33	1.48	0.16
7	45	APE	2.04	1.79	1.78	1.87	0.15
7	45	BHT/BHA	1.64	1.33	1.62	1.53	0.17
7	45	CONTROL	1.72	2.08	2.00	1.93	0.19
7	45	GPE	1.40	1.56	1.29	1.42	0.14
7	45	PPE	1.47	1.64	1.65	1.59	0.10
7	60	APE	1.85	2.26	2.04	2.05	0.21
7	60	BHT/BHA	1.73	1.61	1.48	1.61	0.13
7	60	CONTROL	2.17	2.33	1.87	2.12	0.23
7	60	GPE	1.45	1.72	1.59	1.59	0.14
7	60	PPE	1.94	1.62	1.61	1.72	0.19
14	30	APE	1.91	2.04	2.20	2.05	0.15
14	30	BHT/BHA	1.67	1.69	1.44	1.60	0.14
14	30	CONTROL	2.16	2.24	2.05	2.15	0.10
14	30	GPE	1.67	1.43	1.48	1.53	0.13
14	30	PPE	1.77	1.63	1.74	1.71	0.07
14	45	APE	2.79	2.60	2.67	2.69	0.10
14	45	BHT/BHA	2.00	1.86	2.04	1.97	0.09
14	45	CONTROL	2.87	2.97	2.58	2.81	0.20
14	45	GPE	1.68	1.72	1.57	1.66	0.08
14	45	PPE	2.07	2.35	2.07	2.16	0.16
14	60	APE	2.82	2.60	2.91	2.78	0.16
14	60	BHT/BHA	1.91	2.12	2.26	2.10	0.18
14	60	CONTROL	3.29	2.81	3.16	3.09	0.25
14	60	GPE	1.84	1.93	1.52	1.76	0.22

Day	Temp (°C)	Treatment	Rep 1	Rep 2	Rep 3	Mean	SD
14	60	PPE	2.35	2.60	2.41	2.45	0.13
21	30	APE	2.54	2.39	2.61	2.51	0.11
21	30	BHT/BHA	1.74	1.71	2.04	1.83	0.18
21	30	CONTROL	2.29	2.53	2.63	2.48	0.17
21	30	GPE	1.84	1.69	1.87	1.80	0.10
21	30	PPE	1.95	1.86	2.14	1.98	0.14
21	45	APE	3.20	2.94	3.15	3.10	0.14
21	45	BHT/BHA	2.05	2.24	2.40	2.23	0.18
21	45	CONTROL	3.39	3.74	4.17	3.77	0.39
21	45	GPE	1.95	2.17	1.98	2.03	0.12
21	45	PPE	2.38	2.66	2.68	2.57	0.17
21	60	APE	4.15	4.39	3.97	4.17	0.21
21	60	BHT/BHA	2.83	3.18	2.71	2.91	0.24
21	60	CONTROL	4.73	4.18	4.32	4.41	0.29
21	60	GPE	2.08	2.39	2.34	2.27	0.17
21	60	PPE	3.35	3.00	3.10	3.15	0.18
28	30	APE	2.89	2.80	2.61	2.77	0.14
28	30	BHT/BHA	2.15	2.24	2.39	2.26	0.12
28	30	CONTROL	2.97	3.12	3.44	3.18	0.24
28	30	GPE	1.70	1.97	1.95	1.87	0.15
28	30	PPE	2.54	2.15	2.26	2.32	0.20
28	45	APE	4.22	4.56	4.66	4.48	0.23
28	45	BHT/BHA	3.34	3.02	3.00	3.12	0.19
28	45	CONTROL	4.88	4.75	5.49	5.04	0.40
28	45	GPE	2.60	2.33	2.38	2.44	0.14
28	45	PPE	3.12	3.28	3.54	3.31	0.21
28	60	APE	6.07	6.96	6.29	6.44	0.46
28	60	BHT/BHA	3.60	4.07	3.92	3.86	0.24
28	60	CONTROL	7.02	6.79	6.51	6.77	0.26
28	60	GPE	2.80	2.93	3.09	2.94	0.15
28	60	PPE	4.23	3.93	3.85	4.00	0.20

Day	Temp (°C)	Treatment	Rep 1	Rep 2	Rep 3	Mean	SD
35	30	APE	3.84	3.71	3.57	3.71	0.14
35	30	BHT/BHA	2.60	2.52	2.43	2.52	0.09
35	30	CONTROL	4.42	4.12	4.31	4.28	0.15
35	30	GPE	2.14	2.10	1.88	2.04	0.14
35	30	PPE	2.64	2.78	2.88	2.77	0.12
35	45	APE	6.27	6.33	6.59	6.40	0.17
35	45	BHT/BHA	3.63	3.65	3.96	3.75	0.19
35	45	CONTROL	6.89	6.65	7.32	6.95	0.34
35	45	GPE	2.86	2.59	2.60	2.68	0.15
35	45	PPE	4.29	4.03	3.88	4.07	0.21
35	60	APE	9.71	10.17	9.95	9.94	0.23
35	60	BHT/BHA	5.46	5.55	5.76	5.59	0.15
35	60	CONTROL	9.60	9.97	9.51	9.69	0.24
35	60	GPE	3.44	3.58	3.66	3.56	0.11
35	60	PPE	5.96	5.58	5.67	5.74	0.20

Values are individual replicates (Rep 1–3), followed by mean ± SD (n = 3), for each Treatment × Temperature × Day combination.

Table S4. Complete p-AV data for all treatments, temperatures, and storage days, with individual replicates and summary statistics.

Day	Temp (°C)	Treatment	Rep 1	Rep 2	Rep 3	Mean	SD
7	30	APE	0.69	0.59	0.78	0.69	0.10
7	30	BHT/BHA	0.58	0.58	0.51	0.56	0.04
7	30	CONTROL	0.73	0.86	0.69	0.76	0.09
7	30	GPE	0.57	0.45	0.56	0.53	0.07
7	30	PPE	0.61	0.60	0.50	0.57	0.06
7	45	APE	0.73	0.75	0.85	0.78	0.06
7	45	BHT/BHA	0.73	0.60	0.68	0.67	0.07
7	45	CONTROL	0.84	0.95	0.86	0.88	0.06
7	45	GPE	0.67	0.51	0.68	0.62	0.10
7	45	PPE	0.58	0.74	0.69	0.67	0.08
7	60	APE	1.06	0.80	0.86	0.91	0.14
7	60	BHT/BHA	0.88	0.77	0.70	0.78	0.09
7	60	CONTROL	0.91	1.08	0.93	0.97	0.09
7	60	GPE	0.63	0.67	0.72	0.67	0.05
7	60	PPE	0.81	0.74	0.69	0.75	0.06
14	30	APE	0.75	0.68	0.86	0.76	0.09
14	30	BHT/BHA	0.62	0.67	0.55	0.61	0.06
14	30	CONTROL	0.91	1.07	0.85	0.94	0.11
14	30	GPE	0.61	0.58	0.60	0.60	0.02
14	30	PPE	0.74	0.59	0.70	0.68	0.08
14	45	APE	0.97	0.77	0.89	0.88	0.10
14	45	BHT/BHA	0.76	0.61	0.80	0.72	0.10
14	45	CONTROL	1.06	0.96	1.05	1.02	0.06
14	45	GPE	0.69	0.76	0.58	0.68	0.09
14	45	PPE	0.63	0.87	0.61	0.70	0.14
14	60	APE	1.23	1.16	1.18	1.19	0.04
14	60	BHT/BHA	0.88	0.85	0.77	0.83	0.06
14	60	CONTROL	1.33	1.39	1.17	1.30	0.11
14	60	GPE	0.71	0.74	0.80	0.75	0.05
14	60	PPE	0.97	1.01	0.79	0.92	0.12

Day	Temp (°C)	Treatment	Rep 1	Rep 2	Rep 3	Mean	SD
21	30	APE	0.86	0.84	0.84	0.85	0.01
21	30	BHT/BHA	0.68	0.63	0.85	0.72	0.12
21	30	CONTROL	1.07	1.05	1.27	1.13	0.12
21	30	GPE	0.66	0.64	0.65	0.65	0.01
21	30	PPE	0.67	0.69	0.86	0.74	0.10
21	45	APE	0.94	0.96	1.16	1.02	0.12
21	45	BHT/BHA	0.88	0.98	0.83	0.90	0.08
21	45	CONTROL	1.36	1.12	1.10	1.19	0.14
21	45	GPE	0.71	0.72	0.65	0.69	0.04
21	45	PPE	0.87	0.86	0.93	0.89	0.04
21	60	APE	1.63	1.62	1.46	1.57	0.10
21	60	BHT/BHA	1.28	1.10	1.18	1.19	0.09
21	60	CONTROL	1.70	1.81	1.64	1.72	0.09
21	60	GPE	0.91	0.93	1.03	0.96	0.06
21	60	PPE	1.14	1.26	1.28	1.23	0.08
28	30	APE	1.13	1.27	1.18	1.19	0.07
28	30	BHT/BHA	0.94	0.96	1.10	1.00	0.09
28	30	CONTROL	1.38	1.41	1.49	1.43	0.06
28	30	GPE	0.79	0.72	0.99	0.83	0.14
28	30	PPE	1.08	0.94	1.03	1.02	0.07
28	45	APE	1.50	1.38	1.66	1.51	0.14
28	45	BHT/BHA	1.18	1.14	1.36	1.23	0.12
28	45	CONTROL	1.68	1.71	1.54	1.64	0.09
28	45	GPE	0.96	1.06	0.93	0.98	0.07
28	45	PPE	1.33	1.23	1.13	1.23	0.10
28	60	APE	2.22	2.21	2.01	2.15	0.12
28	60	BHT/BHA	1.56	1.84	1.69	1.70	0.14
28	60	CONTROL	2.28	2.21	2.24	2.24	0.04
28	60	GPE	1.38	1.34	1.27	1.33	0.06
28	60	PPE	1.58	1.75	1.58	1.64	0.10
35	30	APE	1.68	1.60	1.84	1.71	0.12
35	30	BHT/BHA	1.56	1.36	1.34	1.42	0.12

Day	Temp (°C)	Treatment	Rep 1	Rep 2	Rep 3	Mean	SD
35	30	CONTROL	1.97	2.09	1.88	1.98	0.11
35	30	GPE	1.11	1.25	1.24	1.20	0.08
35	30	PPE	1.26	1.50	1.43	1.40	0.12
35	45	APE	2.19	2.33	2.02	2.18	0.16
35	45	BHT/BHA	1.73	1.81	1.55	1.70	0.13
35	45	CONTROL	2.34	2.11	2.32	2.26	0.13
35	45	GPE	1.34	1.31	1.39	1.35	0.04
35	45	PPE	1.76	1.74	1.82	1.77	0.04
35	60	APE	2.76	2.95	2.99	2.90	0.12
35	60	BHT/BHA	2.10	2.40	2.27	2.26	0.15
35	60	CONTROL	3.10	2.93	3.01	3.01	0.09
35	60	GPE	1.73	1.90	1.74	1.79	0.10
35	60	PPE	2.28	2.20	2.07	2.18	0.11

Values are individual replicates (Rep 1–3), followed by mean ± SD (n = 3), for each Treatment × Temperature × Day combination.

Table S5. Complete TOTOX data for all treatments, temperatures, and storage days, with individual replicates and summary statistics.

Day	Temp (°C)	Treatment	Rep 1	Rep 2	Rep 3	Mean	SD
7	30	APE	4.51	3.97	4.38	4.29	0.28
7	30	BHT/BHA	3.84	3.28	3.19	3.44	0.35
7	30	CONTROL	4.23	4.88	4.21	4.44	0.38
7	30	GPE	3.91	3.05	3.28	3.41	0.45
7	30	PPE	3.91	3.52	3.16	3.53	0.38
7	45	APE	4.81	4.33	4.41	4.52	0.26
7	45	BHT/BHA	4.01	3.26	3.92	3.73	0.41
7	45	CONTROL	4.28	5.11	4.86	4.75	0.43
7	45	GPE	3.47	3.63	3.26	3.45	0.19
7	45	PPE	3.52	4.02	3.99	3.84	0.28
7	60	APE	4.76	5.32	4.94	5.01	0.29
7	60	BHT/BHA	4.34	3.99	3.66	4.00	0.34
7	60	CONTROL	5.25	5.74	4.67	5.22	0.54
7	60	GPE	3.53	4.11	3.90	3.85	0.29
7	60	PPE	4.69	3.98	3.91	4.19	0.43
14	30	APE	4.57	4.76	5.26	4.86	0.36
14	30	BHT/BHA	3.96	4.05	3.43	3.81	0.34
14	30	CONTROL	5.23	5.55	4.95	5.24	0.30
14	30	GPE	3.95	3.44	3.56	3.65	0.27
14	30	PPE	4.28	3.85	4.18	4.10	0.23
14	45	APE	6.55	5.97	6.23	6.25	0.29
14	45	BHT/BHA	4.76	4.33	4.88	4.66	0.29
14	45	CONTROL	6.80	6.90	6.21	6.64	0.37
14	45	GPE	4.05	4.20	3.72	3.99	0.25
14	45	PPE	4.77	5.57	4.75	5.03	0.47
14	60	APE	6.87	6.36	7.00	6.74	0.34
14	60	BHT/BHA	4.70	5.09	5.29	5.03	0.30
14	60	CONTROL	7.91	7.01	7.49	7.47	0.45
14	60	GPE	4.39	4.60	3.84	4.28	0.39

Day	Temp (°C)	Treatment	Rep 1	Rep 2	Rep 3	Mean	SD
14	60	PPE	5.67	6.21	5.61	5.83	0.33
21	30	APE	5.94	5.62	6.06	5.87	0.23
21	30	BHT/BHA	4.16	4.05	4.93	4.38	0.48
21	30	CONTROL	5.65	6.11	6.53	6.10	0.44
21	30	GPE	4.34	4.02	4.39	4.25	0.20
21	30	PPE	4.57	4.41	5.14	4.71	0.38
21	45	APE	7.34	6.84	7.46	7.21	0.33
21	45	BHT/BHA	4.98	5.46	5.63	5.36	0.34
21	45	CONTROL	8.14	8.60	9.44	8.73	0.66
21	45	GPE	4.61	5.06	4.61	4.76	0.26
21	45	PPE	5.63	6.18	6.29	6.03	0.35
21	60	APE	9.93	10.40	9.40	9.91	0.50
21	60	BHT/BHA	6.94	7.46	6.60	7.00	0.43
21	60	CONTROL	11.16	10.17	10.28	10.54	0.54
21	60	GPE	5.07	5.71	5.71	5.50	0.37
21	60	PPE	7.84	7.26	7.48	7.53	0.29
28	30	APE	6.91	6.87	6.40	6.73	0.28
28	30	BHT/BHA	5.24	5.44	5.88	5.52	0.33
28	30	CONTROL	7.32	7.65	8.37	7.78	0.54
28	30	GPE	4.19	4.66	4.89	4.58	0.36
28	30	PPE	6.16	5.24	5.55	5.65	0.47
28	45	APE	9.94	10.50	10.98	10.47	0.52
28	45	BHT/BHA	7.86	7.18	7.36	7.47	0.35
28	45	CONTROL	11.44	11.21	12.52	11.72	0.70
28	45	GPE	6.16	5.72	5.69	5.86	0.26
28	45	PPE	7.57	7.79	8.21	7.86	0.33
28	60	APE	14.36	16.13	14.59	15.03	0.96
28	60	BHT/BHA	8.76	9.98	9.53	9.42	0.62
28	60	CONTROL	16.32	15.79	15.26	15.79	0.53
28	60	GPE	6.98	7.20	7.45	7.21	0.24
28	60	PPE	10.04	9.61	9.28	9.64	0.38

Day	Temp (°C)	Treatment	Rep 1	Rep 2	Rep 3	Mean	SD
35	30	APE	9.36	9.02	8.98	9.12	0.21
35	30	BHT/BHA	6.76	6.40	6.20	6.45	0.28
35	30	CONTROL	10.81	10.33	10.50	10.55	0.24
35	30	GPE	5.39	5.45	5.00	5.28	0.24
35	30	PPE	6.54	7.06	7.19	6.93	0.34
35	45	APE	14.73	14.99	15.20	14.97	0.24
35	45	BHT/BHA	8.99	9.11	9.47	9.19	0.25
35	45	CONTROL	16.12	15.41	16.96	16.16	0.78
35	45	GPE	7.06	6.49	6.59	6.71	0.30
35	45	PPE	10.34	9.80	9.58	9.91	0.39
35	60	APE	22.18	23.29	22.89	22.79	0.56
35	60	BHT/BHA	13.02	13.50	13.79	13.44	0.39
35	60	CONTROL	22.30	22.87	22.03	22.40	0.43
35	60	GPE	8.61	9.06	9.06	8.91	0.26
35	60	PPE	14.20	13.36	13.41	13.66	0.47

TOTOX = 2×PV + p-AV, calculated per replicate before averaging. Values are individual replicates (Rep 1–3), followed by mean ± SD (n = 3), for each Treatment × Temperature × Day combination.

Table S6. Complete AV, PV, p-AV, and TOTOX data for butter samples stored under each film treatment at room temperature (RT, 21°C) or in an oven (Oven, 30 °C), with individual replicates and summary statistics.

Treatment	Condition	Rep	AV	PV	p-AV	TOTOX
Initial	RT	1	0.50	0.26	0.89	1.41
Initial	RT	2	0.70	0.36	0.83	1.55
Initial	RT	3	0.60	0.20	0.70	1.10
Initial	RT	Mean	0.60	0.27	0.81	1.35
Initial	RT	SD	0.10	0.08	0.10	0.23
Initial	Oven	1	0.50	0.26	0.87	1.39
Initial	Oven	2	0.70	0.36	0.83	1.55
Initial	Oven	3	0.60	0.20	0.71	1.11
Initial	Oven	Mean	0.60	0.27	0.80	1.35
Initial	Oven	SD	0.10	0.08	0.08	0.22
Control Film	RT	1	1.60	1.86	3.36	7.08
Control Film	RT	2	1.70	1.73	3.53	6.99
Control Film	RT	3	1.55	1.69	3.48	6.86
Control Film	RT	Mean	1.62	1.76	3.46	6.98
Control Film	RT	SD	0.08	0.09	0.09	0.11
Control Film	Oven	1	2.60	2.15	5.25	9.55
Control Film	Oven	2	2.40	2.20	5.10	9.50
Control Film	Oven	3	2.53	2.27	5.17	9.71
Control Film	Oven	Mean	2.51	2.21	5.17	9.59
Control Film	Oven	SD	0.10	0.06	0.08	0.11
Ziplock bag	RT	1	1.10	1.11	3.03	5.25
Ziplock bag	RT	2	1.00	1.23	2.80	5.26
Ziplock bag	RT	3	1.09	1.18	2.96	5.32
Ziplock bag	RT	Mean	1.06	1.17	2.93	5.28
Ziplock bag	RT	SD	0.06	0.06	0.12	0.04
Ziplock bag	Oven	1	1.50	1.61	4.28	7.50
Ziplock bag	Oven	2	1.70	1.63	4.08	7.34
Ziplock bag	Oven	3	1.67	1.71	4.29	7.71
Ziplock bag	Oven	Mean	1.62	1.65	4.22	7.52
Ziplock bag	Oven	SD	0.11	0.05	0.12	0.19
AP-Film	RT	1	1.30	1.41	3.20	6.02
AP-Film	RT	2	1.40	1.47	3.24	6.18
AP-Film	RT	3	1.47	1.63	3.09	6.35
AP-Film	RT	Mean	1.39	1.50	3.18	6.18
AP-Film	RT	SD	0.09	0.11	0.08	0.17
AP-Film	Oven	1	1.80	1.83	4.59	8.25

AP-Film	Oven	2	1.90	1.94	4.67	8.55
AP-Film	Oven	3	1.86	1.85	4.47	8.17
AP-Film	Oven	Mean	1.85	1.87	4.58	8.32
AP-Film	Oven	SD	0.05	0.06	0.10	0.20
PP-Film	RT	1	1.20	1.10	2.77	4.97
PP-Film	RT	2	1.10	1.15	2.91	5.21
PP-Film	RT	3	1.30	1.10	2.99	5.19
PP-Film	RT	Mean	1.20	1.12	2.89	5.12
PP-Film	RT	SD	0.10	0.03	0.11	0.13
PP-Film	Oven	1	1.69	1.69	4.09	7.47
PP-Film	Oven	2	1.70	1.75	4.03	7.53
PP-Film	Oven	3	1.75	1.62	4.22	7.46
PP-Film	Oven	Mean	1.71	1.69	4.11	7.49
PP-Film	Oven	SD	0.03	0.07	0.10	0.04
GP-Film	RT	1	0.80	0.52	2.31	3.35
GP-Film	RT	2	0.70	0.61	2.22	3.44
GP-Film	RT	3	0.86	0.63	2.25	3.51
GP-Film	RT	Mean	0.79	0.59	2.26	3.43
GP-Film	RT	SD	0.08	0.06	0.05	0.08
GP-Film	Oven	1	1.10	1.26	3.37	5.89
GP-Film	Oven	2	1.30	1.15	3.29	5.59
GP-Film	Oven	3	1.20	1.29	3.31	5.89
GP-Film	Oven	Mean	1.20	1.23	3.32	5.79
GP-Film	Oven	SD	0.10	0.07	0.04	0.17

Values are individual replicates (1-3), followed by mean and SD (n = 3), for each Treatment × Condition combination. AV = acid value; PV = peroxide value (meq O_2/kg); p-AV = p-anisidine value; TOTOX = 2×PV + p-AV. Initial corresponds to the day-0 baseline butter, prior to treatment application.
